# Isolating Ligand Steric Effects on Metal Ion Reduction and Gold Nanoparticle Formation Pathways Using Au(I)–Phosphine Complexes

**DOI:** 10.1021/acs.langmuir.6c02684

**Published:** 2026-09-18

**Authors:** Kaveen Tennakoon, Nivedita Shyamsundar, Thomas H. Tugwell, Aklima A. Akhi, Manu Jyothi Ravi, Peng Liu, Shigeru Amemiya, Jill E. Millstone

**Affiliations:** † Department of Chemistry, 6614 University of Pittsburgh, 219 Parkman Avenue, Pittsburgh, Pennsylvania 15260, United States; ‡ Department of Chemical and Petroleum Engineering, University of Pittsburgh, 3700 O’Hara Street, Pittsburgh, Pennsylvania 15261, United States; § Department of Mechanical Engineering, University of Pittsburgh, 3700 O’Hara Street, Pittsburgh, Pennsylvania 15261, United States; ∥ Department of Chemistry, 6612 Carnegie Mellon University, 4400 Fifth Avenue, Pittsburgh, Pennsylvania 15213, United States

## Abstract

Steric effects are widely used to control molecular reactivity, yet their role in nanoparticle (NP) synthesis remains difficult to isolate and leverage because changes in ligand structure often alter both precursor electronic structure and resulting reduction thermodynamics. Here, we use three Au­(I)–phosphine chloride complexes with systematically increasing ligand cone angles to decouple precursor sterics from electronics and determine how steric bulk influences gold NP formation. Density functional theory and cyclic voltammetry show that the three precursors have comparable frontier orbital energies and standard reduction potentials, while their heterogeneous electron-transfer kinetics differ substantially, with increasing steric bulk progressively slowing reduction. When these precursors are used in otherwise identical NP syntheses, the resulting particle populations differ significantly. The least sterically hindered precursor produces the largest, plasmonically active particles, while the most sterically encumbered precursor yields predominantly non-plasmonic cluster-like products. Transmission electron microscopy, extinction spectra, and a custom-built and time-resolved color-intensity analysis are consistent with this relationship indicating that increasing precursor steric bulk both slows NP formation and also suppresses downstream NP growth into larger particles, likely by hindering post-reduction cluster coalescence as evidenced by the appearance of Au cluster populations detected by matrix-assisted laser desorption ionization mass spectrometry. Taken together, these results indicate that precursor sterics impact gold NP formation through dual, but distinguishable roles in metal ion reduction kinetics and also altering downstream NP growth pathways. More broadly, this work establishes precursor sterics as a distinct molecular design parameter for controlling both the kinetics and outcomes of NP synthesis.

## Graphical abstract

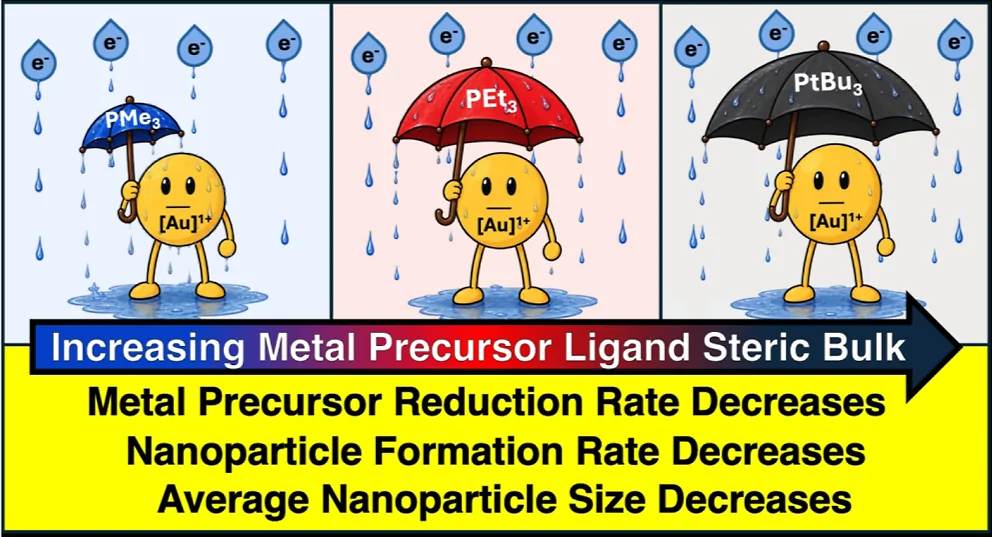

## Introduction

It is an axiom of chemical reasoning that molecular structure dictates both the steric and electronic properties of the molecule and therefore can be used to predict molecular behavior in a given environment or reaction. Well-known examples of these phenomena are found in reactions such as secondary benzene substitutions which may be directed by the electronic and/or steric properties of the initial substituent.^1–3^ Connecting these reagent properties to downstream molecular architecture control facilitates efficient molecular design, diversity, and synthesis. Likewise, developing nanoparticle (NP) syntheses that can leverage the molecular tunability of starting reagents, extends the concept of reagent tailoring to guide not only molecular architectures, but also nanoscale ones.

Thus far, metal NP syntheses have leveraged precursor design primarily to address one of two modes for metal nanostructure control: modulating the reduction kinetics of metal ion precursor(s) and/or the surface reactivity of resulting particles. As a strategy for altering metal ion reduction kinetics, both the metal ion precursor and reducing agents have received significant attention.^4–8^ However, while the role of reagent structure in metal ion reduction kinetics has been widely studied, parsing whether those effects come from steric and/or electronic factors has remained largely opaque, and therefore limited the ability to fully realize the power of molecular reagent design to drive NP formation trajectories.

Yet, given the prominent role of kinetics in determining NP outcomes, one may expect steric modifications to molecular reagents to be a rich tool for NP structural control and diversification. Indeed, steric factors are already well known to be distinct directing agents in a variety of molecular syntheses. For example, Ramadoss et al. showed meta-selective arene C–H borylation by using a “roof” ligand that sterically shields ortho and para positions, guiding activation to the exposed meta site.^9^ Extending these concepts to molecular assembly and materials, a study by Liu et al. showed that steric crowding built directly into tetratopic and ditopic imine-condensation monomers can funnel self-assembly toward large [6 + 12] porous organic cages by disfavoring smaller or interlocked topologies.^10^ In homogeneous catalyst development, Wang et al. added benzosuberyl groups to the ortho position of the N-aryl ring in α-diimine Ni catalysts. This arrangement capped the Ni metal center in a “sandwich-like” fashion, which reduced chain transfer and allowed the production of ultrahigh-molecular-weight polyolefins.^11^ These examples highlight the diversity of roles sterics can play in small molecule reactivity, polymer formation, and even molecular self-assembly. Therefore, there is a strong precedent on which to build similar steric-based reaction controls into nanochemistry.

Motivated by the potential of molecular design to isolate and leverage molecular sterics in metal NP formation and guided by a combination of cyclic voltammetry (CV) and density functional theory (DFT) analyses, here we select three Au­(I) phosphine complexes with ligands of increasing cone angles and find that not only do the sterics of pendant Au­(I) ligands modulate initial metal ion reduction kinetics, as expected, but they also separately participate in directing downstream NP growth pathways. Taken together, the results indicate a rich and multi-modal role for sterics in NP precursory chemistry and, ultimately, NP architectural control.

## Results and Discussion

In order to isolate the role of precursor sterics in metal NP reactions, we needed to control for several confounding factors. First, changes to metal ion ligand environments often change the electronic structure of the metal center. Further, as mentioned above, it is well known that metal ion reduction kinetics influence many aspects of metal NP synthesis, including the resulting NP sizes, size distributions, and shapes in some cases.^12–15^ Therefore, in order to assign observed metal ion reduction kinetics to the impacts of steric hindrance at the metal center, we first analyzed the electronic properties of each complex. Three Au­(I) phosphine complexes were selected to span a range of ligand cone angles while maintaining comparable electronic environments at the metal center, as predicted by DFT analysis (Figure 1). These complexes are chloro­(gold­(I))­trimethylphosphine (AuTMP), cone angle ∼ 118°, chloro­(gold­(I))­triethylphosphine (AuTEtP), cone angle ∼ 132°, and chloro­(gold­(I))­tri-*tert*-butylphosphine (AuT*t*BuP), cone angle ∼ 182°.^16,17^ Table S1 summarizes the DFT-calculated frontier orbital energies (highest occupied molecular orbital (HOMO)/lowest unoccupied molecular orbital (LUMO)) and the Gibbs free energy changes for the reduction of the three precursor complexes. Notably, all three compounds exhibit similar HOMO and LUMO energies (within ∼0.3 eV for HOMOs and ∼0.06 eV for LUMOs), suggesting comparable oxidative reactivity and electron affinities. Consequently, the thermodynamically-driven propensity of each complex to undergo reduction is also predicted to be comparable. These similarities are supported by the calculated Gibbs free energy values for the reduction of each precursor, which are within error for all 3 complexes.

**Figure 1:**
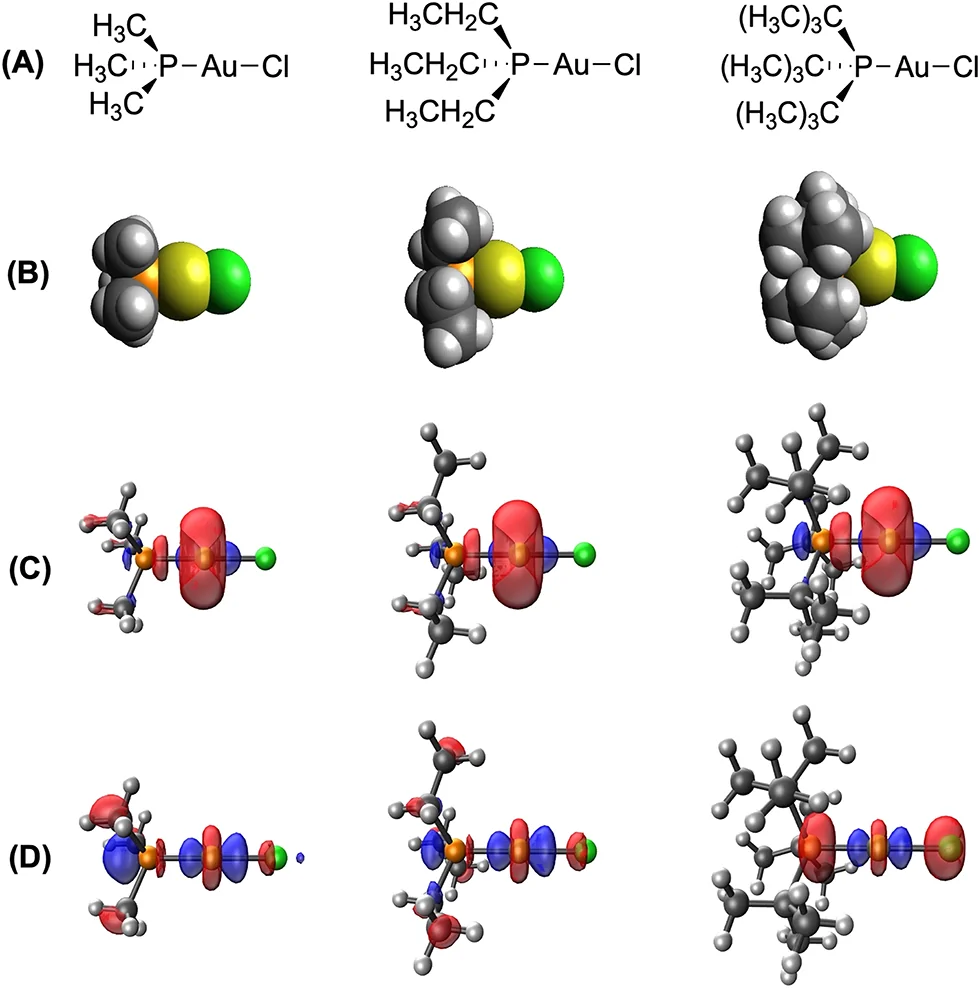
(A) Chemical structures of the three Au­(I)–phosphine chloride complexes studied, (B) space-filled models showing the relative steric bulk of each ligand, (C) LUMO, showing identical antibonding character, (D) HOMO, showing analogous bonding character. Complexes are ordered left to right: AuTMP, AuTEtP, and AuT*t*BuP, representing increasing ligand sterics across the row.

Guided by the predicted similarities in electronic structure between the three complexes, we then measured their electronic properties directly using cyclic voltammetry (CV). In a typical experiment, cyclic voltammograms were recorded using a three-electrode setup (working, counter, and reference electrodes; all Pt) at a scan rate of 2 V/s at room temperature for all three complexes. The resulting voltammograms were then used to quantify the standard reduction potential for each complex. The measured standard reduction potentials of all three complexes remain nearly unchanged across the ligand series (Table 1 and Figure 2), as predicted by their calculated LUMO energies, and their measured HOMO–LUMO gaps also agree well with theory (Table S1). On the other hand, the electron-transfer kinetics differ markedly between the three complexes. The complex with the least sterically demanding ligands, AuTMP displays quasi-reversible redox behavior, while AuTEtP and AuTtBuP show even larger peak separations (Δ*E*_p_) .^18,19^ Such non-Nernstian peak separations are a signature of sluggish electron-transfer kinetics,^20^ where bulky ligands can obstruct the rate of heterogeneous electron transfer, necessitating a greater potential to drive the redox process.^21^ This trend mirrors observations in other systems where added steric encumbrance slows the electron-transfer rate and increases Δ*E*_p_.^22^ Indeed, the evaluated standard heterogeneous electron-transfer rate constant (*k*_e_) for the Au­(I)­LCl/[Au(0)­LCl]^−^ couple (where L is the phosphine ligand) decreases as ligand steric bulk increases, even though the standard potentials (*E*°) are essentially invariant (Table 1 and Supporting Information).^18^ Taken together, these data suggest that steric factors primarily influence the observed redox kinetics (e.g., the distance of electron tunneling between the electrode and the Au complex)) rather than the electronic structure of the complex (i.e., their frontier orbital energies and associated redox properties).^23^

**Table 1:** Electrochemical Parameters for AuTMP, AuTEtP, and AuT*t*BuP Measured by Cyclic Voltammetry, Highlighting the Effect of Sterics on Redox Potential and Electron-Transfer Kinetics

complex	standard redox potential vs Fc/Fc^+^ (V)	peak separation (V)	*k*_e_ (cm/s)	*k*_c_ (s^–1^)
AuTMP	–1.51 ± 0.01	0.23 ± 0.07	(7.55 ± 0.30) × 10^–3^	4.9 ± 0.1
AuTEtP	–1.51 ± 0.02	0.34 ± 0.04	(3.83 ± 0.21) × 10^–3^	4.0 ± 0.2
AuT*t*BuP	–1.50 ± 0.02	0.94 ± 0.03	(8.39 ± 0.80) × 10^–5^	3.5 ± 0.1

**Figure 2:**
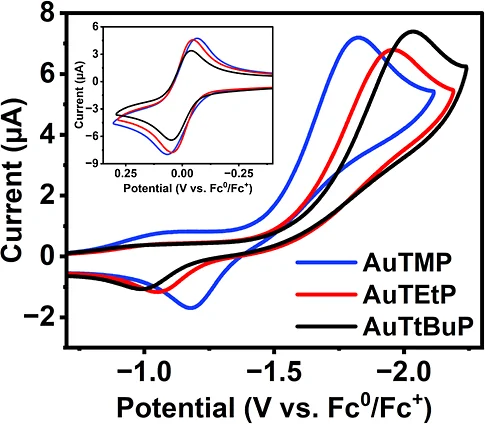
Representative cyclic voltammograms of AuTMP (blue), AuTEtP (red), and AuT*t*BuP (black) complexes corresponding to values reported in Table 1. All voltammograms were collected initially using a Pt electrode reference and then calibrated using ferrocene as an internal reference (Fc^0^/Fc^+^; 0.20 ± 0.05 V vs Pt pseudo-reference under these conditions) in order to account for variability in the Pt surface heterogenetity between experiments (top left inset shows representative Fc^0^/Fc^+^ voltammograms collected from the ferrocene internal reference with each ligand and shows this slight variability associated with Pt reference electrodes).

Interestingly, electrochemical analysis also revealed information about the structural evolution of the metal ion precursor after reduction. At lower scan rates (< 0.1 V/s), no significant anodic peak was observed for any of the complexes. However, as the scan rate increased (> 0.1 V/s), the anodic peak current became more pronounced (Figure S1). This scan-rate-dependent appearance of the oxidation peak is characteristic of an E_i_C_i_-type mechanism, in which an irreversible electron transfer is followed by a subsequent irreversible chemical transformation.^23–25^ In our case, the “missing” anodic peak at slow scan rates implies that once [Au(0)­LCl]^−^ is generated at the electrode, it quickly undergoes a chemical transformation that removes it from the solution near the electrode surface (for example, in a process where the newly created [Au(0)­LCl]^−^ species coalesce into clusters or undergo ligand-associated reactions). At faster scan rates, the time scale of the CV experiment becomes short relative to this chemical change step, allowing some of the [Au(0)­LCl]^−^ produced on the forward (reduction) sweep to survive and become oxidized on the reverse sweep. The net result is the appearance of an anodic wave at high scan rates, as observed here (Figure S1). Using simulations of the obtained voltammograms, the rate constants for this chemical change step (*k*_c_) associated with each complex can be extracted (see Table 1 and associated simulations in the Supporting Information). Consistent with the electron-transfer rates, *k*_c_ also decreases systematically with increasing phosphine ligand cone angle, indicating that the post-electron-transfer process (for example, a coalescence of [Au(0)­LCl]^−^ species) is slower when the ligand is more sterically demanding. These observations suggest that sterically demanding ligands on metal precursors not only slow initial electron-transfer steps during metal ion reduction, but also slow subsequent chemical transformation step(s), such as those related to cluster formation and/or downstream NP formation (*vide infra*).

These electrochemical measurements established that the reduction kinetics of the selected Au­(I)–phosphine complexes are largely governed by the ligand sterics surrounding the metal center rather than differences in their electron affinity or ionization energy as evidenced by similar standard reduction potentials, HOMO–LUMO gaps, and consistent with DFT modeling. With these results in hand, we then tested these complexes in a traditional metal NP synthesis to understand the unique contributions of metal ion precursor sterics to NP outcomes. To investigate the impact of metal ion steric accessibility on final NP architectures, we chose our synthetic conditions and synthetic targets to be well-studied systems so that we could maximize both comparison with previous work and also minimize convolution from additional experimental unknowns.^26–28^ Briefly, in a typical synthesis, Au­(I)–phosphine complexes were dissolved in diethylene glycol (DEG) at a 1:1 molar ratio of metal to ligand, with poly­(vinylpyrrolidone) (PVP, 10 kDa) serving as the stabilizing ligand. Sodium borohydride (NaBH_4_) was then quickly added as the reducing agent, with rapid mixing to ensure uniform reagent distribution. Sodium borohydride was used here based on its ability to reduce all three Au­(I) complexes *E*°_NaBH4_ ∼ −1.92 V vs Fc^0^/Fc^+^, all three complexes *E*° ∼ −1.50 V vs Fc^0^/Fc^+^). After the introduction of sodium borohydride, the reaction mixture is kept at room temperature, undisturbed, and protected from light and dust for 12 h before purification.

Before evaluating the resulting NPs, it was important to establish that the Au­(I)­phosphine complexes remain unchanged when introduced to the NP reaction milieu (for example, possible ligand exchange with the stabilizer, PVP). A series of nuclear magnetic resonance spectroscopy (NMR) experiments on the precursor solutions confirmed that no ligand exchange or coordination between the Au­(I)–phosphine complexes and PVP is detectable (Figure S3). The one-dimensional ^1^H NMR spectra of each complex remained unchanged in the presence of PVP (no peak broadening or shifts),^29^ and diffusion-ordered spectroscopy (DOSY) analysis showed that the diffusion coefficients of the Au complexes were identical to those with and without PVP present (Figure S3). These controls clarify that the complexes modeled and studied by CV are preserved at the outset of the NP formation reaction.

Using the characterized Au­(I) complexes in the described synthesis, we then analyzed both the resulting NP populations and also their evolution at several stages during the reaction. Average final NP diameters follow an inverse order with respect to ligand cone angle: AuTMP yields the largest NPs (3.21 ± 1.00 nm), whereas AuT*t*BuP yields the smallest (1.56 ± 0.58 nm), with AuTEtP yielding NPs with diameters in between the other two (2.61 ± 1.26 nm) (Figure 3). NP size distributions also differ between Au­(I) precursors. NPs synthesized using AuTMP contain a significant fraction of larger NPs (diameter, *d* > 2 nm) and a relatively broad, normal size distribution (standard deviation = 32% of mean diameter). NPs derived from AuT*t*BuP are predominantly small NPs (*d* < 2 nm), the distribution is also normal, but is broader than the AuTMP derived NPs (standard deviation = 40% of mean diameter). Particles synthesized using AuTEtP exhibit a non-Gaussian size distribution (standard deviation = 55% of mean diameter), consistent with the presence of more than one particle population. To interpret what these changes in size and size distribution indicate about the particle growth pathways, we calculated the volume fraction of “small” (*d* < 2 nm) vs “large” (*d* > 2 nm) NPs for NP samples made from each of the three Au­(I) precursors. This analysis suggests that the fraction of Au atoms incorporated into small NPs increases significantly as the precursor cone angle increases (Figure 4). Extinction spectra are also consistent with these results, where NPs formed using the most sterically encumbered Au­(I) complex (AuT*t*BuP), produce the smallest average NP diameters and also exhibit no distinct localized surface plasmon resonance (LSPR) features. Instead, NPs formed using AuT*t*BuP exhibit discrete, molecular-like electronic transitions (440 and 460 nm) consistent with gold clusters (Figure 5).^30–36^ On the other hand, NPs made using AuTMP exhibit a well-defined LSPR peak (λ_max_ = 520 nm).

**Figure 3:**
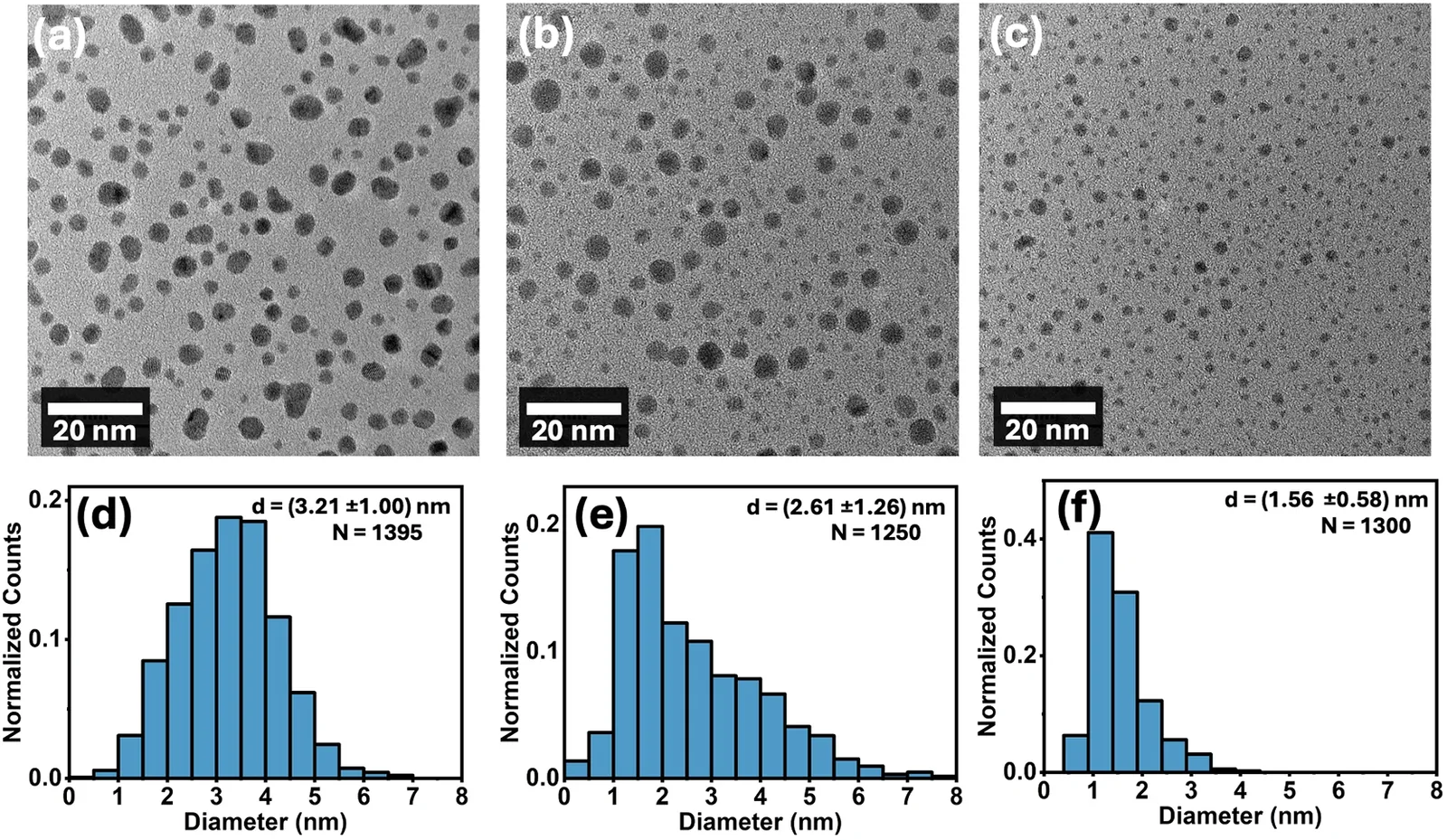
Representative transmission electron microscopy (TEM) images of nanoparticles synthesized from (a) AuTMP, (b) AuTEtP, and (c) AuT*t*BuP, with the corresponding total-count-normalized particle-size distributions shown in (d)–(f), respectively. The data suggest that increasing the steric bulk of the metal precursor is correlated with smaller nanoparticles. Here, *d*represents the mean particle diameter ± standard deviation calculated from the entire population of nanoparticles measured across three independent synthesis trials, while *N* represents the total number of nanoparticles included in the pooled analysis. Particle-size distributions and statistics for each of the three independent synthesis trials are provided in the Supporting Information (Figure S9).

**Figure 4:**
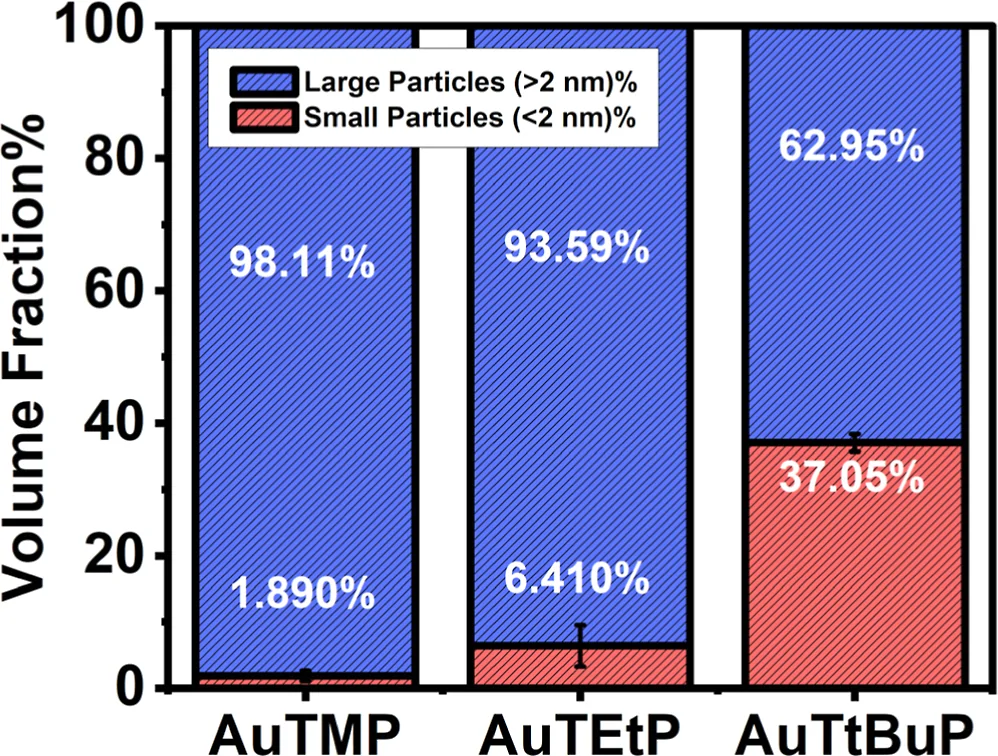
Volume fraction of large (*d* > 2 nm) and small (*d* < 2 nm) nanoparticles formed from AuTMP, AuTEtP, and AuT*t*BuP. Greater steric bulk of metal precursors results in a higher proportion of small particles, highlighting the impact of the precursor structure on NP size and size distribution.

**Figure 5:**
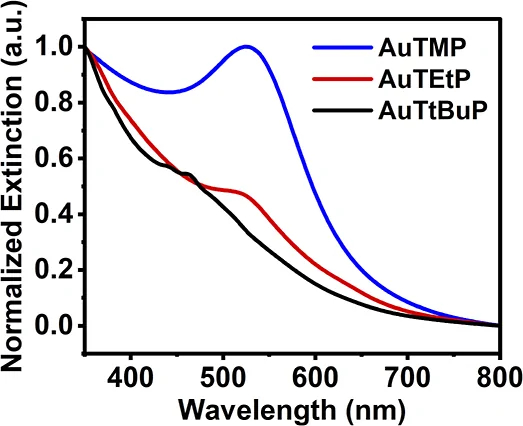
Extinction spectra of the final nanoparticles formed from AuTMP (blue), AuTEtP (red), and AuT*t*BuP (black) after 12 h, consistent with TEM-observed size trends, where Au­(I) complexes with smaller ligands were associated with the formation of larger, plasmonic nanoparticles.

The kinetics of NP formation using each complex were monitored by tracking the optical features of the forming AuNPs. However, when analyzing the kinetics of NP formation, these experiments encountered limitations with extinction spectroscopy because the optical density of the resulting solutions was too high to obtain physically meaningful data from the extinction spectra.^37–39^ Therefore, we developed a color-intensity monitoring method using videos collected on a Canon EOS R8 Mirrorless camera combined with a custom video analysis code (see Supporting Information for code workflow, procedure details, and validation controls, Figure S5). The video analysis approach enables semi-quantitative, real-time observation of NP formation without requiring optically transparent or translucent solutions. Using this approach, we leverage the visible-light LSPR of the forming AuNPs, in concert with the lack of LSPR from smaller Au NPs (*d* < 2 nm, which do not exhibit an observable LSPR band in the visible region and instead show discrete, molecular-like absorptions in the UV–vis region,^34,40^ *vide supra*). Therefore, because the visible color intensity of the reaction solution is proportional to the concentration of larger NPs in solution,^41–43^ tracking the development of solution color intensity during the reaction provides a measure of NP formation kinetics (Figure S7). First derivative plots of color intensity vs time (Figure 6) illustrate NP formation rates. All three systems exhibit an initial spike in the rate of color change that decays to undetectable as the reaction completes. Reactions using AuTMP reaches a maximum color-change rate of (380 ± 1) × 10^–3^ s^–1^ and this peak is reached within 8 s of NaBH_4_ addition, after which the color-change rate falls rapidly, consistent with NP formation that quickly exhausts available precursor species. AuTEtP takes approximately 3 times longer (24 s) to reach a slightly lower peak rate of color change (160 ± 9) × 10^–3^ s^–1,^ and the rate of color change in this reaction returns to zero by ∼40 s, suggesting a still fast, but more extended NP formation process. Strikingly, when AuT*t*BuP is used as the metal ion precursor, the maximum NP formation rate is almost 2 orders of magnitude slower (1.69 ± 0.38) × 10^–3^ s^–1^ and it takes 10 times longer to reach this maximum rate (150 s) compared to syntheses using AuTMP as the precursor. Further, in reactions using AuT*t*BuP, the solution color change rate remains non-zero for over 1800 s (∼30 min) before no further changes are detected. Taken together, these results suggest that increasing ligand steric bulk in the Au­(I) precursor species can impose a successive kinetic barrier to both metal ion reduction as well as particle formation processes.

**Figure 6:**
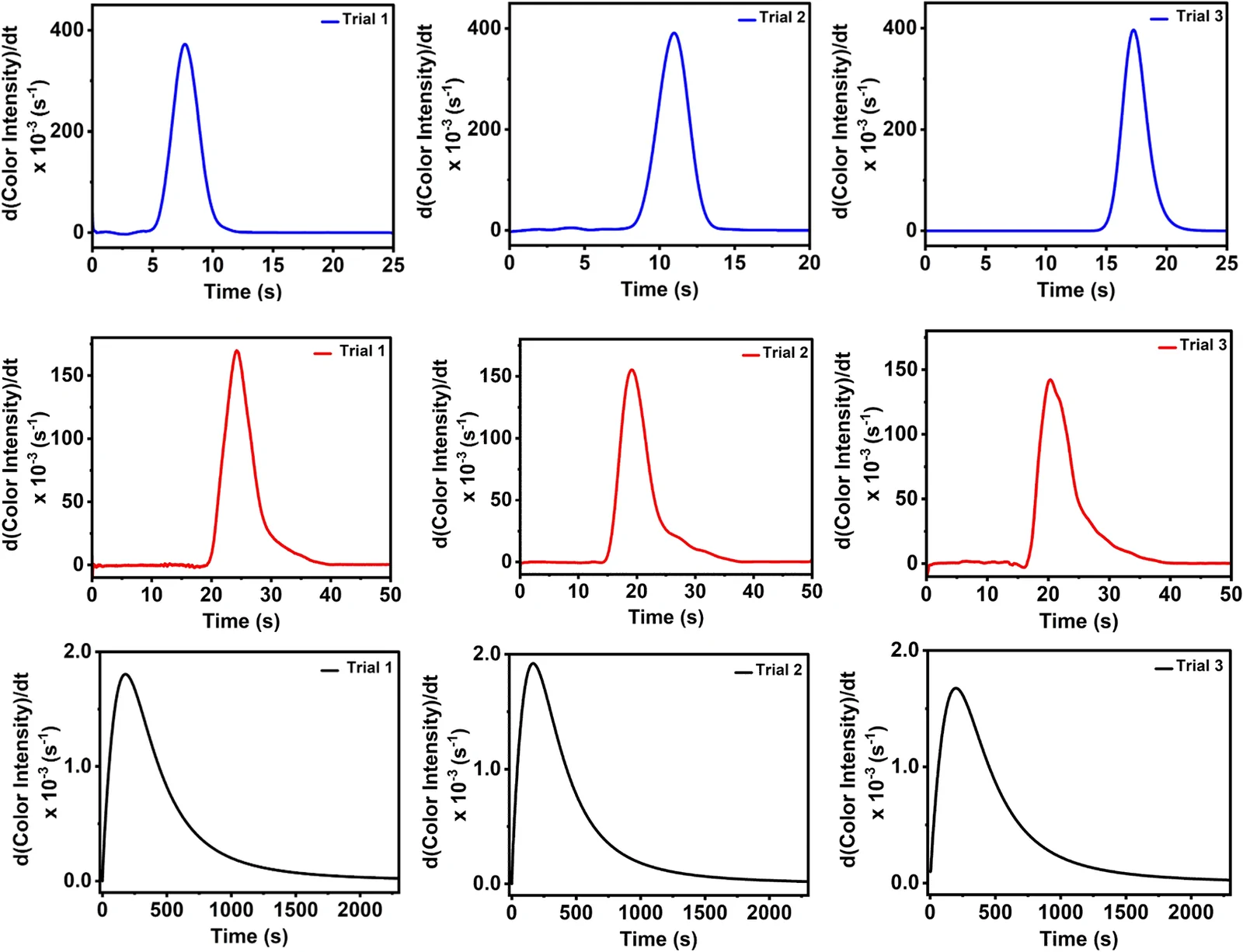
Rate of change in color intensity vs time plots for the three Au­(I)–phosphine precursors, obtained from three replicate videos per system (columns: Trials 1–3). The top row shows AuTMP (blue), the middle row AuTEtP (red), and the bottom row AuT*t*BuP (black) (*N. B.* Minor variations in the time axis between panels arise from small differences in how the start of each reaction (*t* = 0) was defined when cropping the original videos, rather than from differences in the underlying kinetics.).

Previous NP formation models suggest that rapid supersaturation in monomer leads to a short (or “burst”) nucleation event,^44–48^ whereas a sustained monomer supply results in continuous NP nucleation concurrent with particle growth.^49–52^ In these classic nucleation theory-based models, a lower instantaneous monomer concentration should also depress homogeneous nucleation and impact both the number of nuclei formed and how large each grows. Indeed, there are several examples of NP syntheses showing that a reduced NP nucleation rate leads to fewer particles that grow larger, if other factors do not intervene.^46,47,53^ Conversely, using more reactive conditions tends to cause rapid monomer supersaturation and a “burst” of NP formation, yielding many nuclei that compete for available monomer and thus exhibit smaller sizes and less size heterogeneity.^44,48^ A priori, one might anticipate that our results would align with this traditional model: AuTMP, characterized by rapid metal ion reduction kinetics, produces a high concentration of monomer species quickly and therefore yields rapid NP nucleation, monomer consumption, and ultimately relatively small NP sizes and a narrow NP size distribution. On the other hand, AuT*t*BuP would generate fewer nuclei over longer periods, allowing formed NPs to grow and/or ripen, leading to both larger NP sizes and broader size distributions.

However, our results show the opposite NP size trends with metal ion precursor reduction kinetics (Figures 3 and 5), which suggests the involvement of an additional directing process­(es) alongside metal ion reduction kinetics.^54,55^ For example, when the initial metal ion precursor is reduced, our CV analyses indicate that they produce a distinct, ligated-Au(0) product. We hypothesize that once the Au­(I)–phosphine complex is reduced, it quickly reacts to form stable gold phosphine clusters, resulting in a ligated-Au(0) product,^56–58^ as suggested by the E_i_C_i_-mechanism. These clusters may then undergo coalescence and/or atom-by-atom growth to form the final, observed NPs, and consistently, ligand sterics are known to influence the efficiency of such coalescence processes (Scheme 1).^46,59–62^

**Scheme 1:**
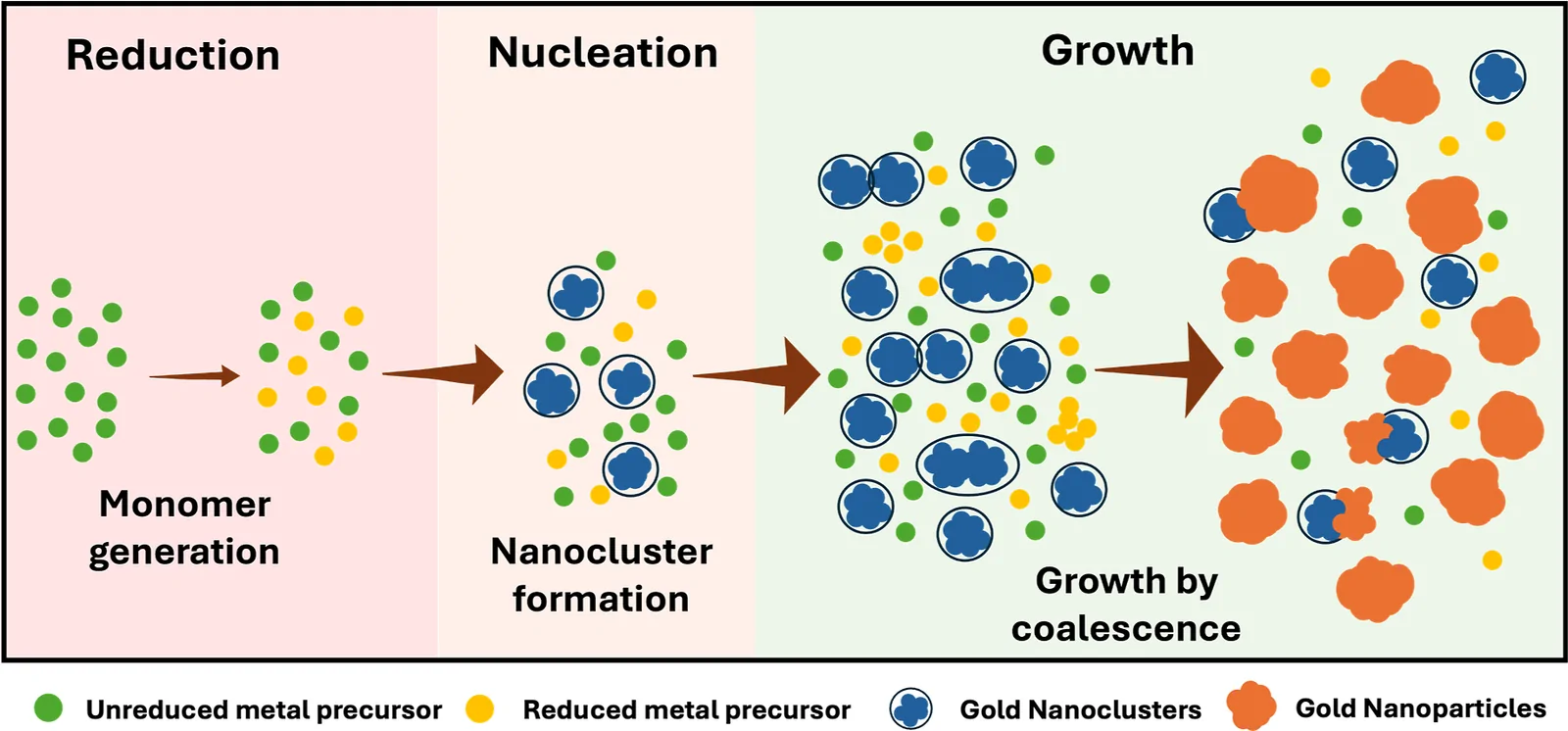
Proposed Pathway of NP Formation^a^

a Following metal ion reduction and monomer generation, nanoclusters form, as evidenced by optical signatures in extinction spectra and mass spectrometry, followed by the appearance of plasmonically-active NPs, and likely due to cluster coalescence (*vide infra*).

If such clusters are formed, as indicated by the scan rate-dependent CV analysis (Figure S1), we reasoned that they should be detectable via mass spectrometry analysis. Matrix-assisted laser desorption/ionization (MALDI) mass spectrometry analysis of the reaction after 12 h of reaction time indicates the presence of small gold clusters in all three systems but at dramatically varying concentrations (see Supporting Information; Figure S4, Table S2). In MALDI spectra from the AuTMP-derived reaction milieu, the primary detectable feature is centered at the Au_101_ regime, but the peak exhibits a very low signal-to-noise ratio, consistent with a low final abundance of discrete cluster intermediates and/or poor detection of high-mass species. The weak AuTMP cluster signal is consistent with clusters that may initially form but have already coalesced into larger, less easily detected species (e.g., large NPs).^63^ In contrast, MALDI analysis of the AuTEtP-based reaction milieu shows a detectable population of clusters (mass centered at approximately ∼Au_75_) with moderate intensity, while the most sterically hindered AuT*t*BuP-derived NP system exhibits three well-resolved low-mass cluster populations (Au_38_, Au_54,_ and Au_68_). Taken together, the size and relative presence of metal clusters observed in the mass spectrometry experiments support the proposed formation pathways, with the sterically hindered AuT*t*BuP-based synthesis producing a population of stable cluster species (also observed in the extinctions spectra and indicated in TEM), and the least sterically hindered precursor, AuTMP, leading to rapidly consumed precursor species that yield the largest particles observed out of the three precursors.

Although this study focuses on Au­(I)–phosphine precursors, the results suggest a broader design principle for NP synthesis where molecular precursor sterics, specifically, can be used to regulate metal ion reduction kinetics and the lifetime of early-stage growth intermediates. This concept is relevant to a variety of metallic and multi-metallic NP syntheses that rely on metal ion reduction kinetics to regulate the size, shape, and/or atomic arrangement of the final NP structures.^64–66^ Specifically, our results suggest that slowing precursor reduction under mild, ambient-temperature conditions can extend the time window over which transient species, such as reduced monomers, small clusters, and/or ligand-bound intermediates, can be detected by optical spectroscopy, electrochemistry, mass spectrometry, or in situ methods. This principle may be especially useful for multimetallic NP syntheses, where the kinetics of metal incorporation can significantly impact final NP architectures and downstream properties.^5,15,67^

## Conclusion

In summary, the systematic study of Au­(I)–phosphine complexes suggests that increasing steric hindrance of the metal ion precursor impacts NP formation at both nucleation and growth stages, distinct from any changes to the thermodynamics of the redox process. Cyclic voltammetry and extinction analyses illustrate that greater steric bulk significantly slows heterogeneous electron-transfer kinetics, thus delaying monomer generation and subsequent nucleation events. This kinetic suppression is manifested in prolonged NP formation times. TEM characterization and particle growth analysis support the conclusion that sterically encumbered ligands also impact NP growth processes, possibly by inhibiting cluster coalescence, and yield consistently smaller and more uniform particle products. Taken together, these results isolate a distinct role of precursor sterics in gold NP outcomes, and further investigations, including in situ spectroscopic studies and computational modeling, will be powerful complements to uncover mechanistic details of the bond-breaking and bond-making steps associated with these processes. Importantly, this study points to an expanded role for precursor sterics as a nanochemistry synthesis tool where they can be used to tune both metal ion reduction and particle formation trajectories orthogonally to the precursor electronic structure. Ultimately, leveraging precursor sterics offers a rich suite of approaches to both manipulating NP formation outcomes and deepening our fundamental understanding of NP synthesis in general.

## Experimental Section

### Materials and Reagents

AuTMP (99.0%), AuTEtP (97.0%), AuT*t*BuP, electrochemical-grade tetrabutylammonium hexafluorophosphate ([*n*Bu_4_N]­[PF_6_], 99.9%) and *trans*-2-[3-(4-*tert*-butylphenyl)-2-methyl-2-propenylidene]­malononitrile (DCTB, 99.0%) were obtained from Sigma-Aldrich (St. Louis, MO). Sodium borohydride (NaBH_4_, ≥98%), and polyvinylpyrrolidone (PVP, average molecular weight (MW) ∼10 kDa) were purchased from Sigma-Aldrich (St. Louis, MO). Poly­(ethylene glycol) methyl ether thiol (mPEGSH, average MW = 1 kDa and 350 Da) was purchased from Laysan Bio, Inc. (Arab, AL). Deuterated dimethyl sulfoxide (DMSO-*d*_6_, 99.9%) was purchased from Cambridge Isotope Laboratories, Inc. (Tewksbury, MA). Diethylene glycol (DEG, 99%) and electrochemical-grade anhydrous dichloromethane (DCM, 99.99%) were used as solvents for all NP syntheses and spectroscopic measurements and were obtained from Sigma-Aldrich (St. Louis, MO). All reagents were used as received unless otherwise noted. NANOpure (Thermo Scientific, ≥18.2 MΩ·cm) water was used to prepare all aqueous solutions.

### Synthesis of Gold Nanoparticles

NPs were synthesized by chemical reduction of the corresponding Au­(I)–phosphine chloride precursor in DEG. In a typical synthesis, 5.00 mL of a 4.0 mM Au­(I)­LCl solution in DEG was combined with 2.50 mL of an 8.0 mM PVP solution in DEG (PVP, average MW = 10 kDa) and 1.50 mL of pure DEG in a 20 mL scintillation vial, corresponding to a 1:1 metal-to-ligand ratio. The reaction mixture was stirred for 10 min at room temperature. NP formation was initiated by rapidly injecting 1.00 mL of freshly prepared 0.2 M NaBH_4_ in DEG into the Au­(I) precursor/PVP solution while vortexing the reaction vial for rapid mixing. Upon injection of the reducing agent, the reaction mixture turned brownish-yellow, indicating NP formation. After vortexing, the reaction was left undisturbed at room temperature for 12 h. Following particle growth, 2.0 mL aliquots of each reaction mixture were transferred to 50 mL Falcon tubes, and 2.0 mL of 1 mM mPEGSH (1 kDa) was added to achieve a metal-to-ligand ratio of 1:0.5. The mixtures were then allowed to undergo ligand exchange with constant agitation for an additional 2 h prior to purification. This ligand-exchange step was necessary to improve particle stability, because as-prepared particles tended to precipitate during purification.

### Purification

Following NP growth and ligand exchange, each sample was purified using 10 kDa molecular-weight-cutoff centrifugal filters (Amicon Ultra centrifugal filter units, EMD Millipore). A 0.25 mL aliquot of each reaction mixture was placed into the filter unit containing Nanopure water and centrifuged at 4000 rcf for 10 min (Eppendorf centrifuge 5804 with swing bucket rotor A-4-44, Hauppauge, NY). This process was repeated twice to concentrate the sample. Identical procedures were followed for AuTMP, AuTEtP, and AuT*t*BuP NP samples.

### UV–Vis Extinction Spectroscopy

Following ligand exchange and purification, the washed NP samples were resuspended in Nanopure water for optical characterization. Extinction spectra were collected using an Agilent Cary 600 NIR UV–vis–NIR spectrophotometer. Samples were loaded into 1 mm path-length quartz cuvettes and evaluated using a 600 nm/min step size. Nanopure water was used as the reference for all measurements.

### Video Analysis

Reaction progress was first monitored by video capture using a Canon EOS R8 mirrorless camera operated at 180 fps. The videos were cropped to a defined region of interest and analyzed in Python using a home-built code to extract the mean gray intensity and its time derivative (see Supporting Information for additional details). Briefly, the Python code imports all necessary functions and software and defines a new function that is called upon for video analysis. This function (1) creates lists that will contain the gray intensity values, (2) sets frame rate in video, (3) processes gray intensity in videos by converting RGB into single-channel grayscale and calculating the mean intensity across each frame, (4) converts frame number to time in seconds, (5) inverts and normalizes intensity data based on observed min/max gray values to relate decrease in intensity to disappearance of color, (6) exports intensity data to Excel, (7) plots average intensity, (8) determines rate of change in intensity, (9) exports rate of change data to Excel, and (10) plots rate of change. Intensity and rate-of-change data are subsequently analyzed by using the data processing software of choice.

### Video Analysis Validation Controls

The video analysis method was validated against UV–vis–NIR spectroscopy Cary 600 NIR spectrophotometer (Agilent, Inc., Santa Clara, CA) and the well-studied “blue bottle” experiment.^68^ In this system, methylene blue cycles between its blue and colorless forms: reduction by dextrose renders the solution colorless, while exposure to oxygen restores the blue color. In this study, the blue-to-colorless transition was specifically monitored, corresponding to a disappearance of color. The reaction was monitored at 664 nm, the λ_max_ of methylene blue, and 6 measurements/min were performed. For comparison with video color analysis, the methylene blue redox reaction was recorded in a scintillation vial. In both methods, the time interval exhibiting the greatest change in absorbance or color intensity was isolated, and the corresponding negative slope (rate of change) was calculated. The slopes obtained from extinction spectroscopy and color analyses were in good agreement (within ∼5%, Figure S5).

### Cyclic Voltammetry

CV measurements were carried out using a CH Instruments CHI600E potentiostat in anhydrous dichloromethane (DCM, 99.99%) with 0.1 M [*n*Bu_4_N]­[PF_6_] as the supporting electrolyte. All three electrodes-working, reference, and counter-were platinum. The Au­(I)­LCl complexes were dissolved to a concentration of 1.00 mM in DCM. CV scans were recorded at variable scan rates (0.1–10 V/s). After the collection of the CVs for each Au­(I)­LCl complex, ferrocene was added directly to the same electrochemical solution as an internal standard. Specifically, 0.93 mg of ferrocene was added to achieve a final concentration of 1.00 mM, and an oxidative scan was then collected to measure the Fc^0^/Fc^+^ redox couple. The standard potential of the Fc^0^/Fc^+^ couple was determined and used to calibrate the potential axis for each CV, such that all reported potentials are referenced relative to Fc^0^/Fc^+^. Fc^0^/Fc^+^ appeared at 0.20 ± 0.05 V vs Pt pseudo-reference under these conditions. Digital simulations performed using CHI600E software were used to estimate the standard reduction potential, heterogeneous standard electron-transfer rate constant (*k*_e_), and chemical-step rate constant (*k*_c_) (see Supporting Information for additional details).

Before each CV measurement, the Pt working electrode was cleaned by mechanical polishing with 50 nm of Al_2_O_3_ polishing particles (Buehler) on a 73 mm polishing pad (Buehler). The electrode was manually polished using a figure-eight motion for 60 cycles and then sonicated in Nanopure water for 1 min to remove residual alumina particles. After sonication, the electrode was rinsed thoroughly with additional Nanopure water, acetone, and finally with the solvent used for the electrochemical experiment. The electrode was then briefly air-dried before use, and CV data were collected immediately after cleaning to minimize changes in electrode surface conditions that could affect heterogeneous electron-transfer kinetics.

### ^1^H NMR Spectroscopy

Each complex was dissolved in DMSO-*d*_6_, and 500 μL of the sample was transferred to 5 mm NMR tubes (Wilmad, Inc.) ^1^H NMR spectra were recorded on a Bruker 600 Ultrashield magnet with AVANCE III 600 Console (Bruker Biospin, Billerica, MA) at 298 K using deuterated dimethyl sulfoxide (DMSO-*d*_6_, 99.9%) (Cambridge Isotope Laboratories, Inc.) as the solvent unless otherwise noted. For all experiments, a *T*_1_ time of 1.00 s was used, and 16 scans were collected.

### NMR Analysis of Au­(I)–Phosphine Precursors Before and After Addition of Poly­(vinylpyrrolidone)

Each Au­(I)–phosphine chloride complex, AuTMP, AuTEtP, and AuT*t*BuP, was analyzed both in the absence and presence of excess poly­(vinylpyrrolidone) (PVP) to determine whether precursor coordination or ligand exchange occurred prior to reduction (Figure S3). For the PVP-containing samples, the polymer was added (metal/ligand = 1:1) directly to the precursor solution and mixed prior to spectrum acquisition.

### Diffusion-Ordered Spectroscopy Acquisition Parameters

Two-dimensional DOSY spectra were acquired using a stimulated-echo pulsed-field-gradient sequence on the same Bruker 600 MHz instrument. Measurements were carried out at 298 K and linearly ramped from 5 to 95% of the maximum gradient output. The diffusion delay (Δ) was set to 100 ms, the relaxation delay (δ) was 1500 s, and 16 scans were collected per increment. Diffusion coefficients were extracted by fitting the signal attenuation as a function of gradient strength using the Stejskal–Tanner equation.

### MALDI-Time-of-Flight Mass Spectrometry

Sample preparation for MALDI followed the same steps as gold NP synthesis and purification. However, during the ligand exchange step, 350 Da PESGH was used instead of 1000 Da PEGSH to obtain a lower cluster mass, which enhances detection. Each precursor was then mixed with *trans*-2-[3-(4-*tert*-Butylphenyl)-2-methyl-2-propenylidene]­malononitrile (DCTB) at a 1:500 (Au/matrix) ratio. Subsequently, 1 μL of each sample was dropped onto a Bruker MALDI plate and analyzed using the Bruker Daltonics UltrafleXtreme MALDI TOF. All MALDI-TOF experiments were conducted in linear mode with a pulsed nitrogen laser (337 nm). Each mass spectrum was acquired with 100 laser shots.^69^

### Transmission Electron Microscopy

Following purification, 8.0 μL of each AuNP sample was deposited onto carbon-coated copper TEM grids (Ted Pella, Inc.) by drop-casting. The grids were allowed to air-dry and were subsequently dried under vacuum prior to imaging. TEM characterization was performed using a Hitachi H9500 Environmental TEM operated at 300 kV (Nanoscale Fabrication and Characterization Facility, Petersen Institute of Nanoscience and Engineering, University of Pittsburgh). NP size distributions were obtained from TEM micrographs by measuring at least 400 particles per sample using ImageJ 1.47d (National Institutes of Health, USA). Resulting histograms (e.g., Figures 3 and S9) and volume-fraction calculations were carried out from the fitted size distributions, as described in the Supporting Information.

## Supplementary Material



## Notes

Details of computational methods and precursor electronic structure analysis; electrochemical calculations and scan-rate studies; NMR and DOSY characterization details; additional MALDI analysis; video-analysis validation and kinetic plot details; and volume-fraction calculations from TEM data (PDF)

## Abbreviations

AuTEtP: chloro­(gold­(I))­triethylphosphine
AuTMP: chloro­(gold­(I))­trimethylphosphine
AuT*t*BuP: chloro­(gold­(I))­tri-*tert*-butylphosphine
NP/NPs: nanoparticle/nanoparticles
DFT: density functional theory
CV: cyclic voltammetry
DOSY: diffusion-ordered spectroscopy
NMR: nuclear magnetic resonance
MALDI: matrix-assisted laser desorption/ionization
TEM: transmission electron microscopy
UV–vis: ultraviolet–visible spectroscopy
LSPR: localized surface plasmon resonance
PVP: poly­(vinylpyrrolidone)
DEG: diethylene glycol
DCM: dichloromethane
DMSO-*d*_6_: deuterated dimethyl sulfoxide
NaBH_4_: sodium borohydride
Fc/Fc^+^: ferrocene/ferrocenium
HOMO: highest occupied molecular orbital
LUMO: lowest unoccupied molecular orbital
E_i_C_i_: irreversible electron transfer followed by an irreversible chemical step
*k*_e_: heterogeneous standard electron-transfer rate constant
*k*_c_: chemical-step rate constant
