# Supplementary material for: Isolating Ligand Steric Effects on Metal Ion Reduction and Gold Nanoparticle Formation Pathways Using Au(I)–Phosphine Complexes
Source: Langmuir. 2026 Sep 18;42(38):27902–12. doi: 10.1021/acs.langmuir.6c02684 (PMC13641092; doi:10.1021/acs.langmuir.6c02684)
Supplement: Supplementary file 1 [file la6c02684_si_001.pdf]

Supporting Information

Isolating Ligand Steric Effects on Metal Ion Reduction and Gold Nanoparticle Formation Pathways Using Au(I)–Phosphine Complexes

Kaveen Tennakoon, [a] *Nivedita Shyamsundar,*[a,d] *Thomas H. Tugwell,* [a] *Aklima A. Akhi,* [a] *Manu Jyothi Ravi,* [a] *Peng Liu,* [a] *Shigeru Amemiya,* [a] *Jill E. Millstone**[,abc]

[a]Department of Chemistry, University of Pittsburgh, 219 Parkman Ave, Pittsburgh, PA 15260

[b]Department of Chemical and Petroleum Engineering and [c]Department of Mechanical Engineering, University of Pittsburgh, 3700 O'Hara Street, Pittsburgh, PA 15261

[d]Department of Chemistry, Carnegie Mellon University, 4400 Fifth Avenue, Pittsburgh, PA, 15213

*Email: jem210@pitt.edu

Table of Contents

S1. COMPUTATIONAL ANALYSIS 3

S2. ELECTROCHEMICAL ANALYSIS.. 5

S2.1 Scan Rate Analysis 5

S2.2. CV Simulations 5

S3. NMR ANALYSIS 6

S4. MALDI ANALYSIS 7

S5. VIDEO ANALYSIS 8

S5.1 Video Code Validation 8

S5.2 Color Intensity Data Acquisition 10

S6. TEM ANALYSIS 12

S6.1 Independent Trials TEM Images and Histograms 12

S6.2 Volume Fraction Calculations 14

S1. COMPUTATIONAL ANALYSIS

Table S1. Calculated HOMO–LUMO energies, HOMO/LUMO energy gaps (ΔE), molar volumes (V_{molar}), and Gibbs free energy of reduction ($\Delta G_{Reduction}$) for AuTMP, AuTEtP, and AuTtBuP complexes in DCM and DEG solvents.

Solvent	Complex	HOMO (eV)	LUMO (eV)	|ΔE| (eV)	V_{molar} (bohr^3/mol)	$\Delta G_{Reduction}$ (kcal/mol)
DCM	AuTMP	-7.42	-0.97	6.45	1151.09	-13.94
	AuTEtP	-7.39	-0.91	6.48	1815.62	-16.76
	AuTtBuP	-7.14	-0.96	6.18	2552.90	-15.13
DEG	AuTMP	-7.37	-0.96	6.41	1151.09	-18.46
	AuTEtP	-7.36	-0.92	6.45	1582.85	-20.71
	AuTtBuP	-7.11	-0.98	6.13	2534.71	-19.83

All density functional theory (DFT) calculations were performed using Gaussian 16.[1] Geometry optimizations of the Au(I)–phosphine chloride complexes (AuTMP, AuTEtP, and AuTtBuP) were carried out in the gas phase using the B3LYP[2,3] exchange–correlation functional with Grimme's D3[4] empirical dispersion correction. Gold atoms were described using the Stuttgart–Dresden (SDD)[5] effective core potential and associated basis set, while all other atoms (C, H, P, Cl) were treated with the 6-31G(d) basis set.[6] Vibrational frequency calculations were performed at the same level of theory to confirm that all optimized structures correspond to true local minima and to obtain zero-point energy corrections.

Single-point energy calculations were subsequently performed on the optimized geometries using the M06 functional,[7] which has been shown to reliably describe transition-metal thermochemistry and noncovalent interactions. For these calculations, the SDD basis set was retained for gold, while the 6-311+G(d,p) basis set[8] was employed for all other atoms. Solvent

effects were incorporated using the SMD continuum solvation model,[9] with dichloromethane (DCM) and diethylene glycol (DEG) used to represent the electrochemical and nanoparticle synthesis environments, respectively.

Frontier molecular orbital energies (HOMO and LUMO), HOMO–LUMO gaps, and orbital distributions were obtained from the M06/SDD–6-311+G(d,p)/SMD single-point energy calculations. Molecular volumes were computed using the volume=tight keyword at the M06/SDD–6-311+G(d,p)/SMD level and were used for comparison across the ligand series. Gibbs free energies of reduction ($\Delta G_{\text{Reduction}}$) were calculated by comparing the free energies of the Au(I)LCl complexes and their corresponding reduced $[\text{Au(0)LCl}]^-$ species within the same solvent environment. All reported thermodynamic quantities correspond to 298.15 K.

This computational framework was used to assess whether changes in ligand steric bulk alter the electronic structure of the Au(I) center or primarily influence kinetic accessibility and steric shielding, thereby enabling direct comparison with experimental cyclic voltammetry and nanoparticle formation data.

S2. ELECTROCHEMICAL ANALYSIS

S2.1 Scan Rate Analysis

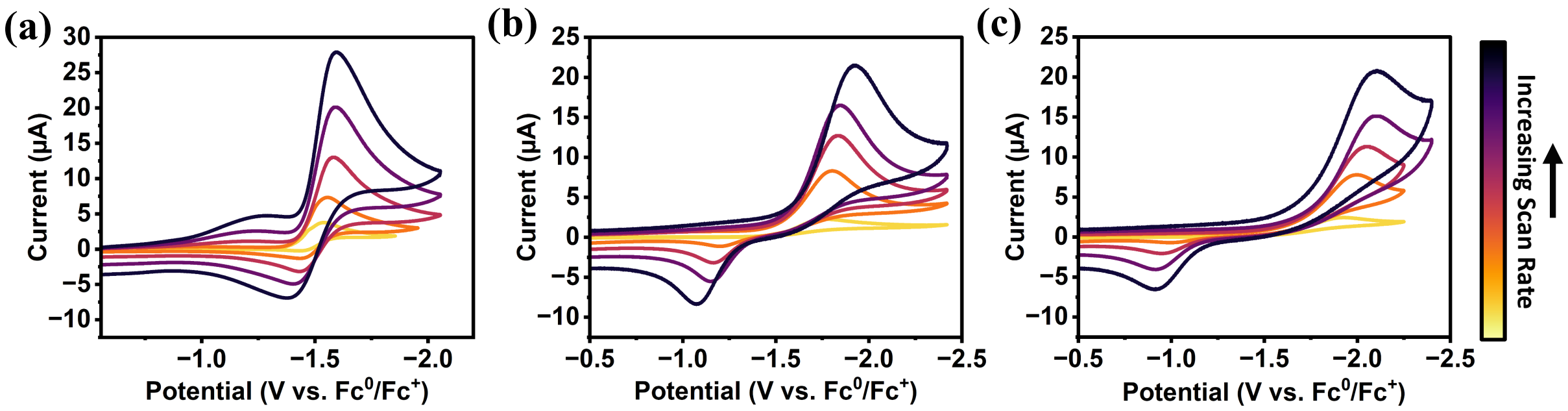

Figure S1. Cyclic voltammograms recorded at increasing scan rates of 0.1, 1.0 , 2.0, 5.0, and 10.0 V/s for the three gold phosphine complexes: (a) AuTMP, (b) AuTEtP, and (c) AuTtBuP. Each graph illustrates the evolution of electrochemical behavior as a function of scan rate for the respective complex.

S2.2. CV Simulations

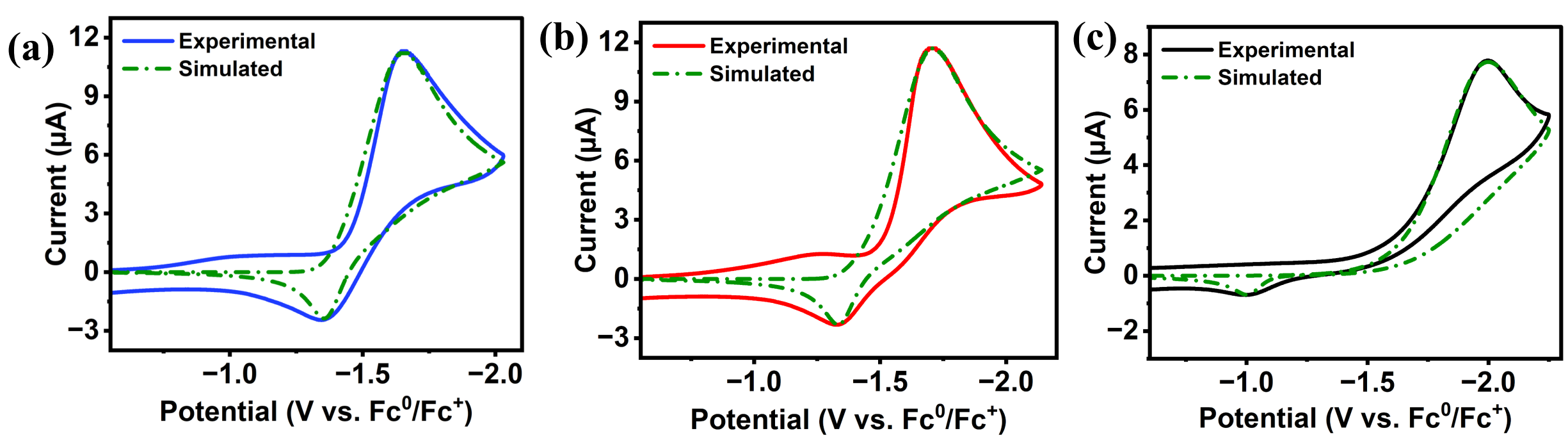

Figure S2. Comparison of the experimental and simulated cyclic voltammograms for the reduction of the gold phosphine complexes: (a) AuTMP (blue, α = 0.33), (b) AuTEtP (red, α = 0.40), and (c) AuTtBuP (black, α = 0.28). Solid lines represent the experimental data, and green dashed lines represent the simulated voltammograms. The simulated CVs were constructed using CHI600E software and were used to estimate the standard reduction potential (E°), heterogeneous standard electron-transfer rate constant (k_e), and chemical-step rate constant (k_c) for each complex. The simulations replicate the overall shape of the cathodic and anodic features, supporting the kinetic analysis of the electrochemical reduction process with the E_iC_i-type reduction mechanism.

S3. NMR ANALYSIS

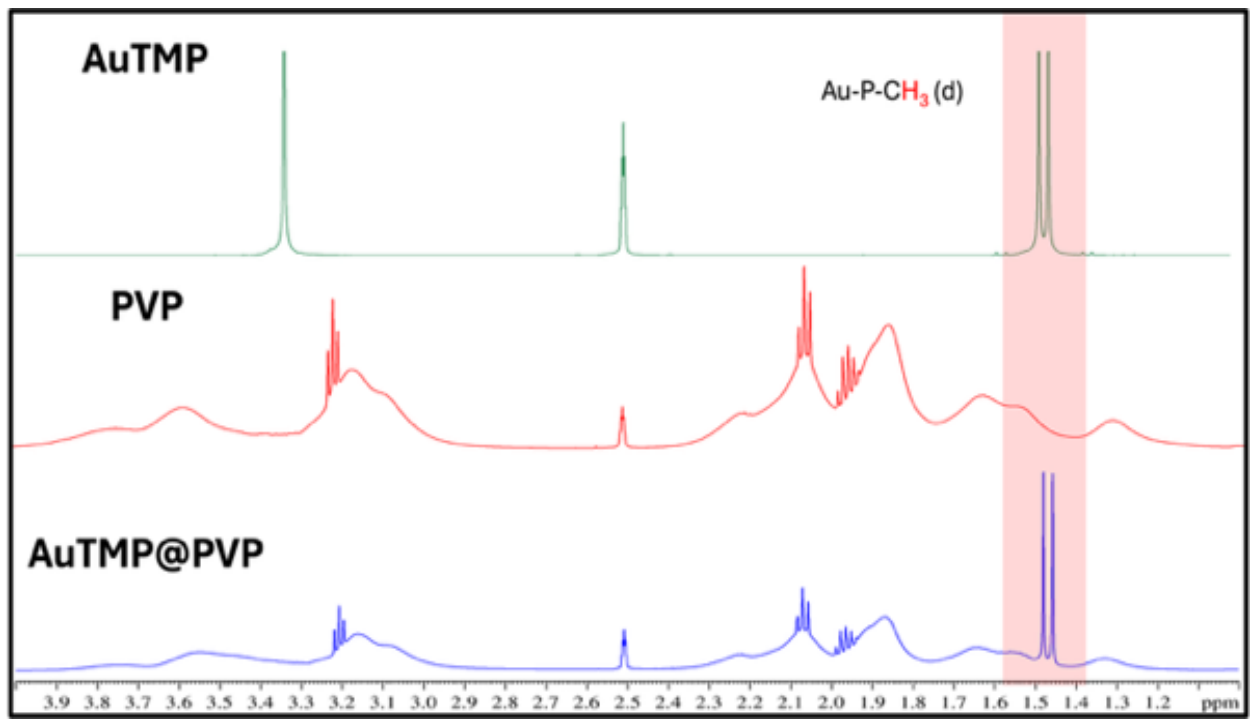

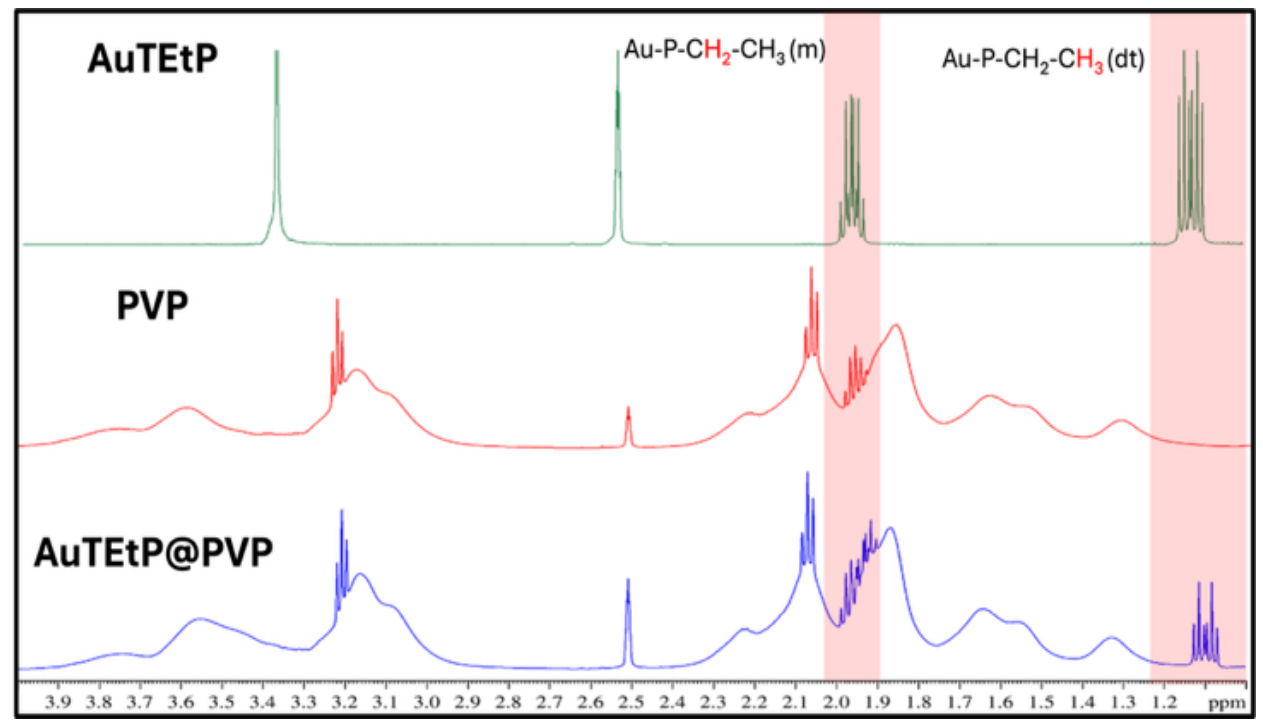

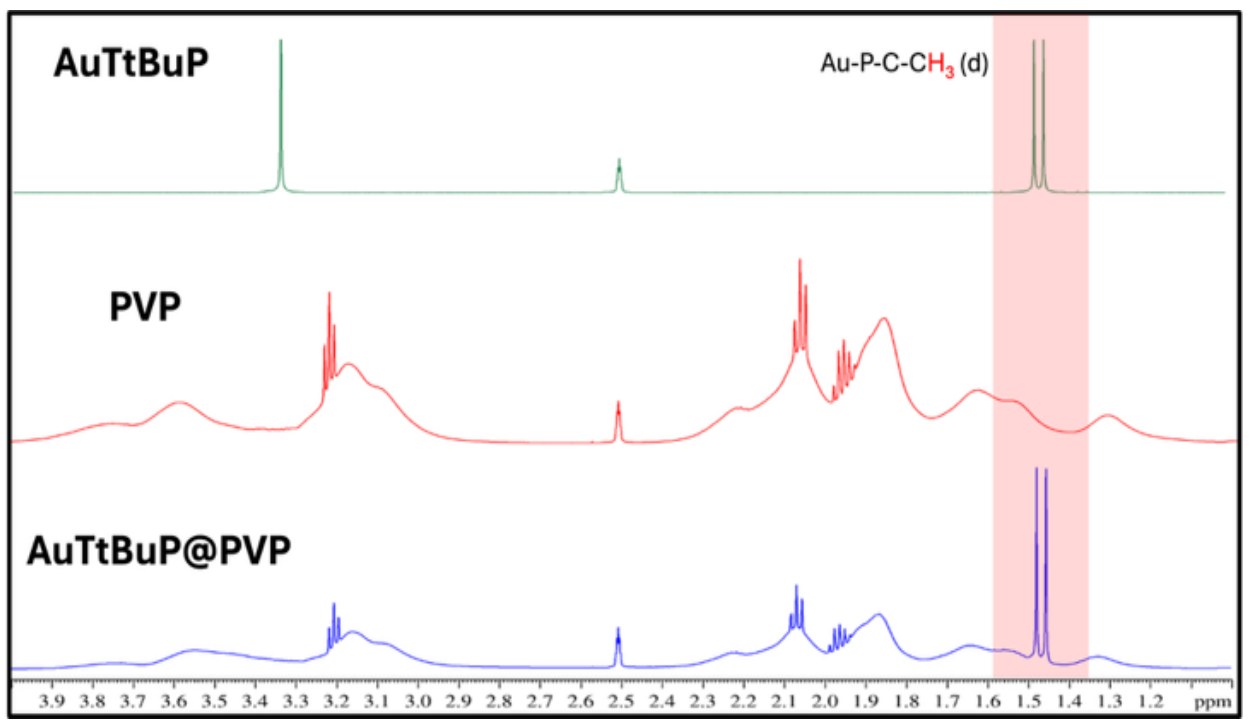

Complex	Diffusion Coefficient (m^2/s)	
	Before adding PVP	After adding PVP
AuTMP	3.24×10^{-10}	3.17×10^{-10}
AuTEtP	3.05×10^{-10}	2.95×10^{-10}
AuTtBuP	2.51×10^{-10}	2.47×10^{-10}

Figure S3. ^{1}H NMR spectra of AuTMP, AuTEtP, and AuTtBuP before and after mixing with PVP, compared with pure PVP. The table summarizes the diffusion coefficients of each complex before and after the addition of PVP.

AuTMP: ^{1}H NMR (600 MHz, DMSO-d_6): δ 1.63 (d, J_{PH}= 9.0 Hz, 9H)

AuTEtP: ^{1}H NMR (600 MHz, DMSO-d_6): δ 1.93 (m, 6H), δ 1.10 (dt, J_{PH} = 9.0 Hz, J_{HH} = 7.0 Hz, 9H)

AuTtBuP: ^{1}H NMR (600 MHz, DMSO-d_6): δ 1.46 (d, J_{PH} = 9.0 Hz, 27H)

S4. MALDI ANALYSIS

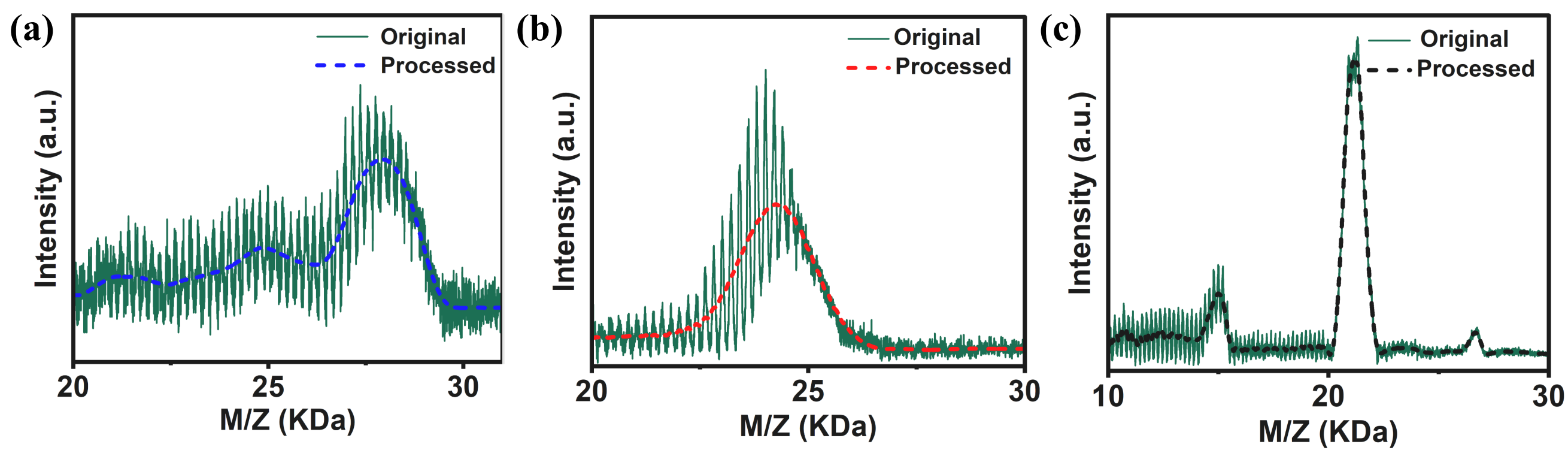

Figure S4. MALDI mass spectra of the gold phosphine samples after 12 h of particle growth are shown as raw (green) and smoothed signals (dashed lines) using the Savitzky–Golay method. (a) AuTMP (b) AuTEtP (c) AuTtBuP.

Table S2. Summary of the MALDI mass spectral analysis of gold phosphine cluster samples, including the observed m/z values, estimated signal-to-noise (S/N) ratios, and proposed assignment of observed mass peaks. Proposed assignments are given for AuTMP, AuTEtP, and AuTtBuP-derived species containing mPEGSH (350 Da), phosphine, and chloride ligands.

Complex	m/z (Da)	S/N Ratio	Suspected Clusters Formular
AuTMP	~27,000	~9	$Au_{101}(PEGSH)_x(PMe_3)_{24-x}Cl_y$
AuTEtP	~24,000	~36	$Au_{75}(PEGSH)_x(PEt_3)_{30-x}Cl_y$ [10,11]
AuTtBuP	~15,000	~7	$Au_{38}(PEGSH)_x(PtBu_3)_{22-x}Cl_y$
	~21,000	~108	$Au_{54}(PEGSH)_x(PtBu_3)_{30-x}Cl_y$ [12]
	~27,000	~10	$Au_{68}(PEGSH)_x(PtBu_3)_{36-x}Cl_y$

S5. VIDEO ANALYSIS

S5.1 Video Code Validation

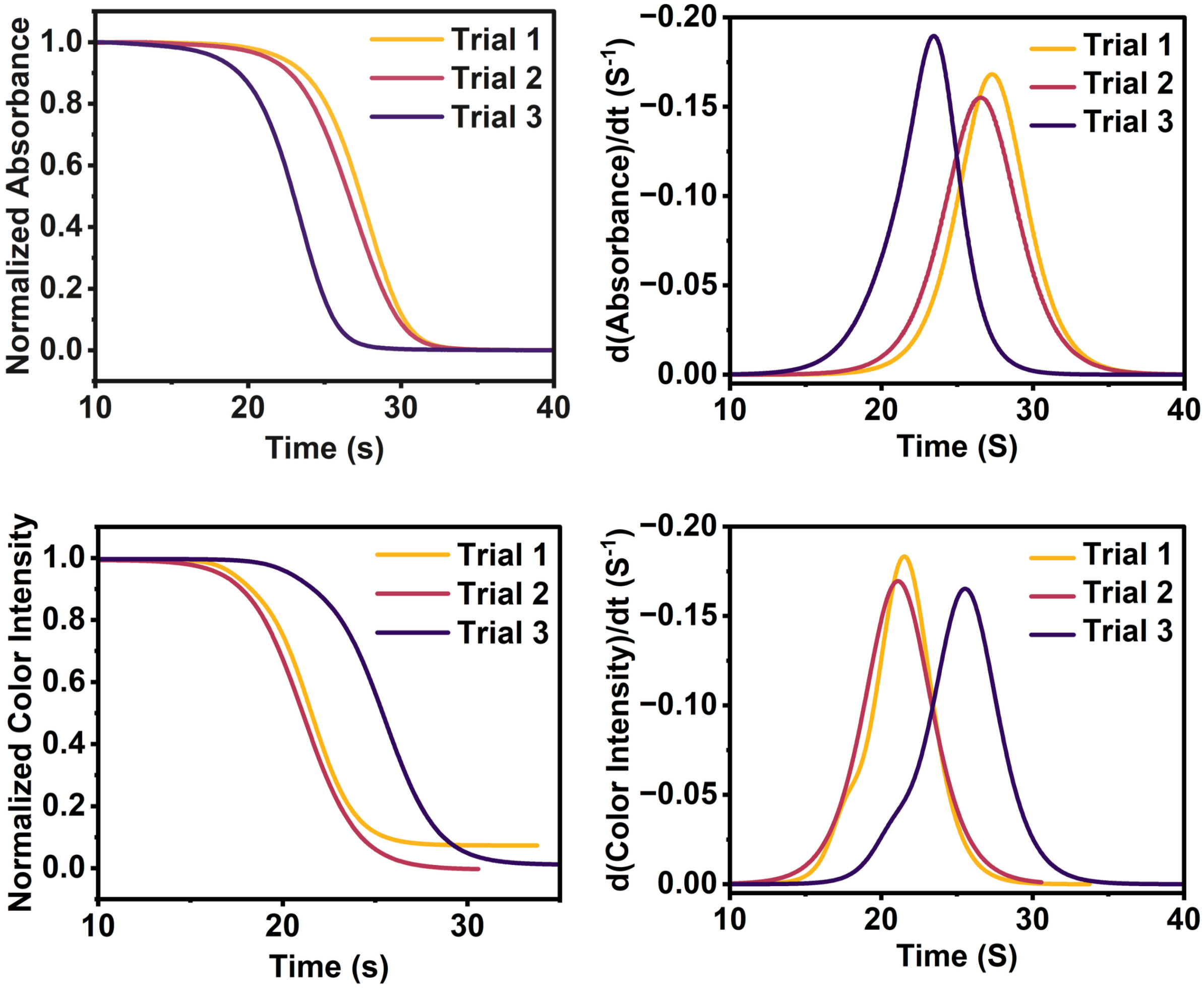

Figure S5. Validation of the custom video-analysis code using the methylene blue-bottle reaction benchmark. Top panels show the absorbance analysis, including normalized absorbance vs. time (left) and the corresponding rate of absorbance change vs. time (right) for three independent trials. The bottom panels show the video analysis, including normalized color intensity vs. time (left) and the corresponding rate of color change vs. time (right) for the same three independent trials. In both methods, the temporal profiles and maximum rates of signal change are in close agreement, supporting the use of the video-analysis code as a reliable method for tracking solution-phase color changes. (Variations in the time axis between trials reflect differences in oxygen exposure, which determine the onset of color fading; however, the similar slopes across trials indicate that the duration and maximum rate of color change remain consistent)

To validate the custom code used for video-based reaction analysis, we selected the methylene blue "blue bottle" reaction as a model system because it exhibits a distinct, delayed color change from dark blue to clear that is readily monitored by optical methods[13–15]. The blue bottle reaction is a well-established color-changing redox system, and methylene-blue-based processes are commonly followed by UV-vis absorbance spectroscopy, making it a suitable benchmark for comparison. Under identical reaction conditions, the reaction was first monitored by UV-vis absorbance spectroscopy to obtain the maximum rate of absorbance change. The same reaction was then recorded using a Canon R8 camera at 180 fps, and the videos were analyzed with custom-built code to extract time-dependent color-intensity data, from which the maximum rate of color change was calculated (**Figure S5**). Prior peer-reviewed studies have shown that digital-image-derived color metrics can correlate closely with UV–vis absorbance and can reproduce equivalent kinetic information when imaging conditions are controlled.[16–18] Based on three independent trials for each method, the maximum rate obtained by UV–vis analysis was $-(0.177 \pm 0.018)\ s^{-1}$, whereas the value obtained from video analysis was $-(0.168 \pm 0.001)\ s^{-1}$. The difference between these values is within ~5% and falls within the experimental uncertainty of the UV–vis measurement, indicating that the video-analysis workflow reproduces the kinetic trend measured by the standard spectroscopic method.[14]

S5.2 Color Intensity Data Acquisition

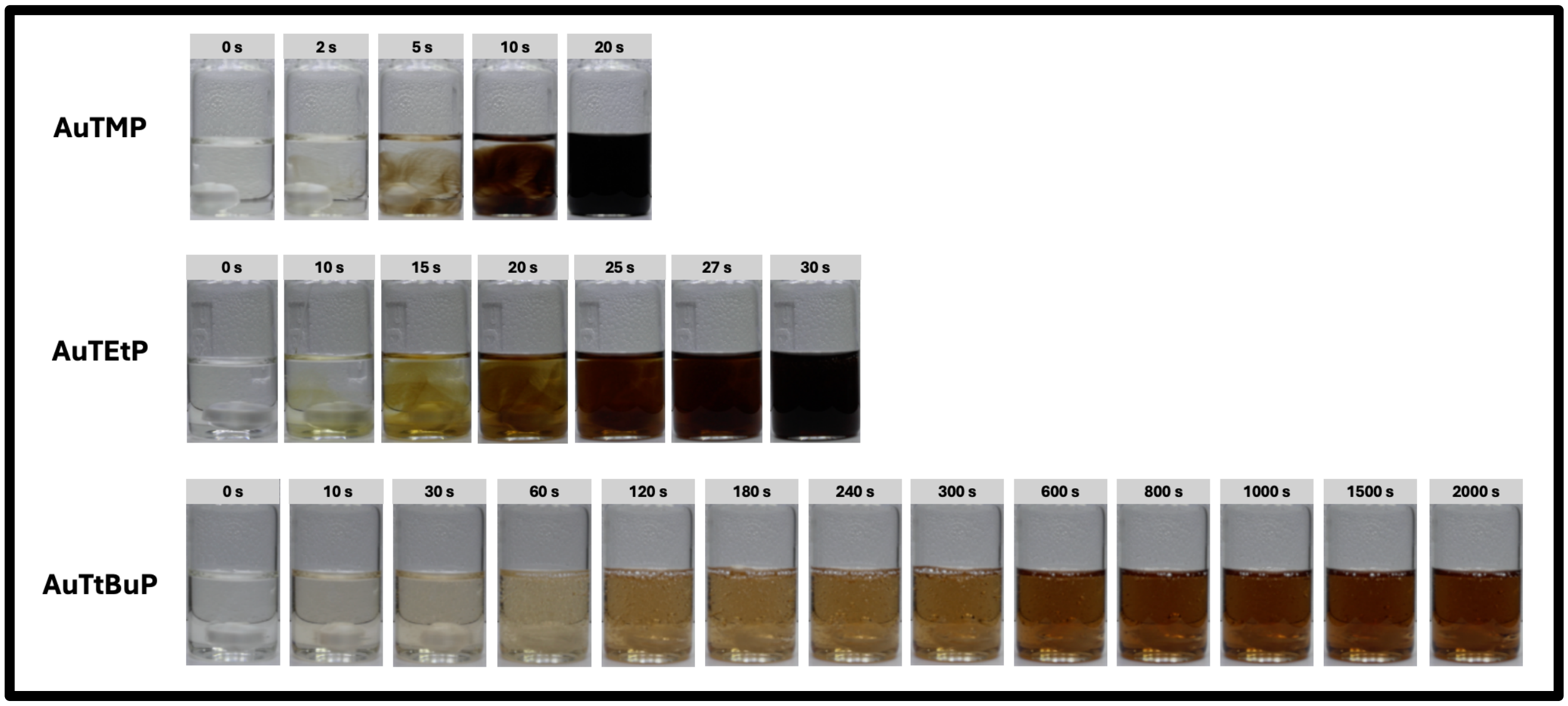

Figure S6. Optical images showing reaction color evolution during reduction of the gold phosphine complexes. AuTMP undergoes the most rapid color change, reaching a dark solution within 20s, whereas AuTEtP shows an intermediate rate and darkens over 30s. In contrast, AuTtBuP undergoes a much slower transformation, with a gradual development of NPs over 2000 s. The images qualitatively illustrate the strong ligand-dependent differences in reduction and nanoparticle formation kinetics.

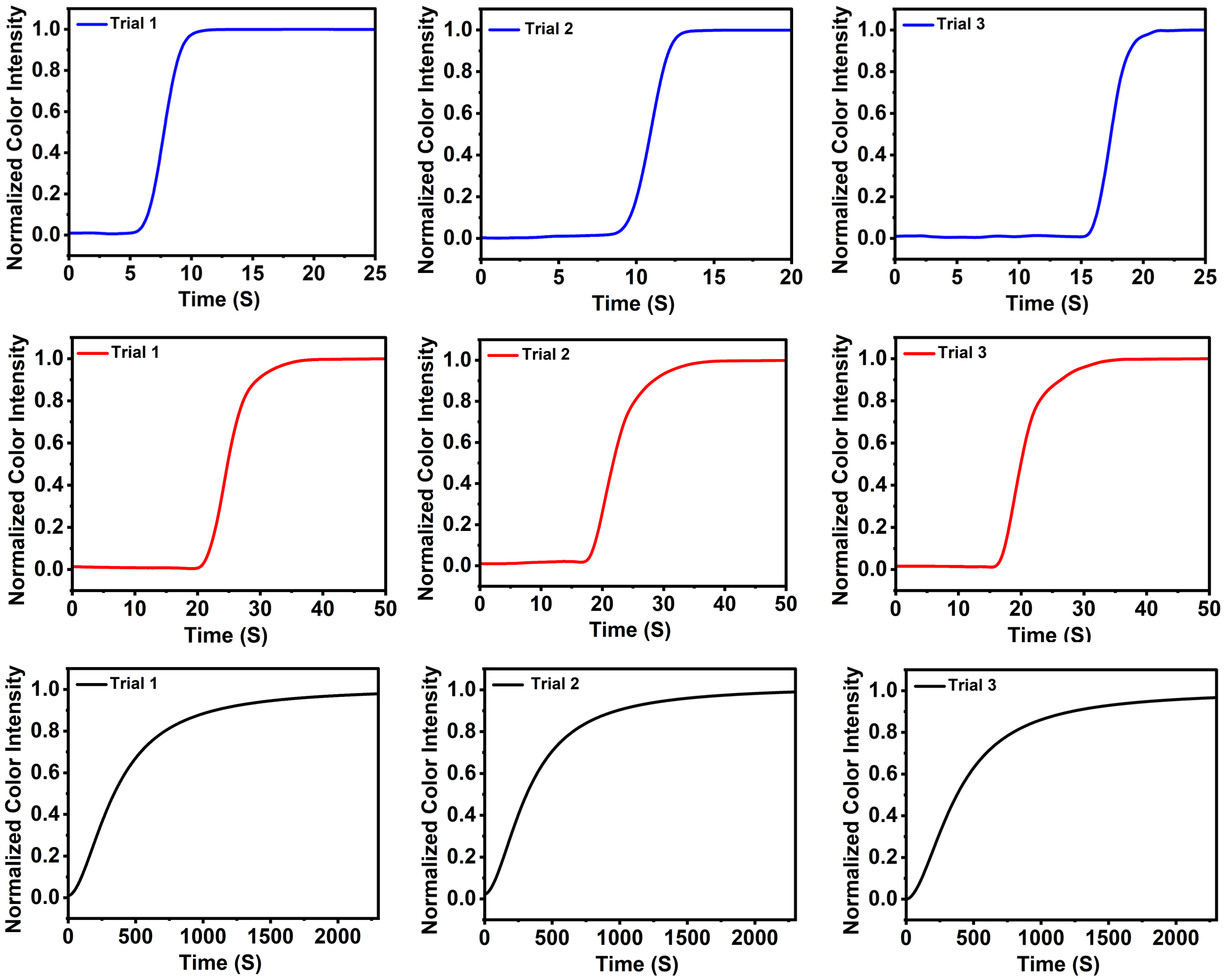

Figure S7. Normalized color intensity vs, time for the three Au(I)–phosphine precursors, obtained using three replicate videos per system (columns: Trials 1–3). The top row shows AuTMP (Blue), the middle row AuTEtP (Red), and the bottom row AuTtBuP (Black). (Variations in the time axis between panels arise from small differences in how the start of each reaction (t = 0) was defined when cropping the original videos, rather than from differences in the underlying kinetics.)

S6. TEM ANALYSIS

S6.1 Independent Trials TEM Images and Histograms

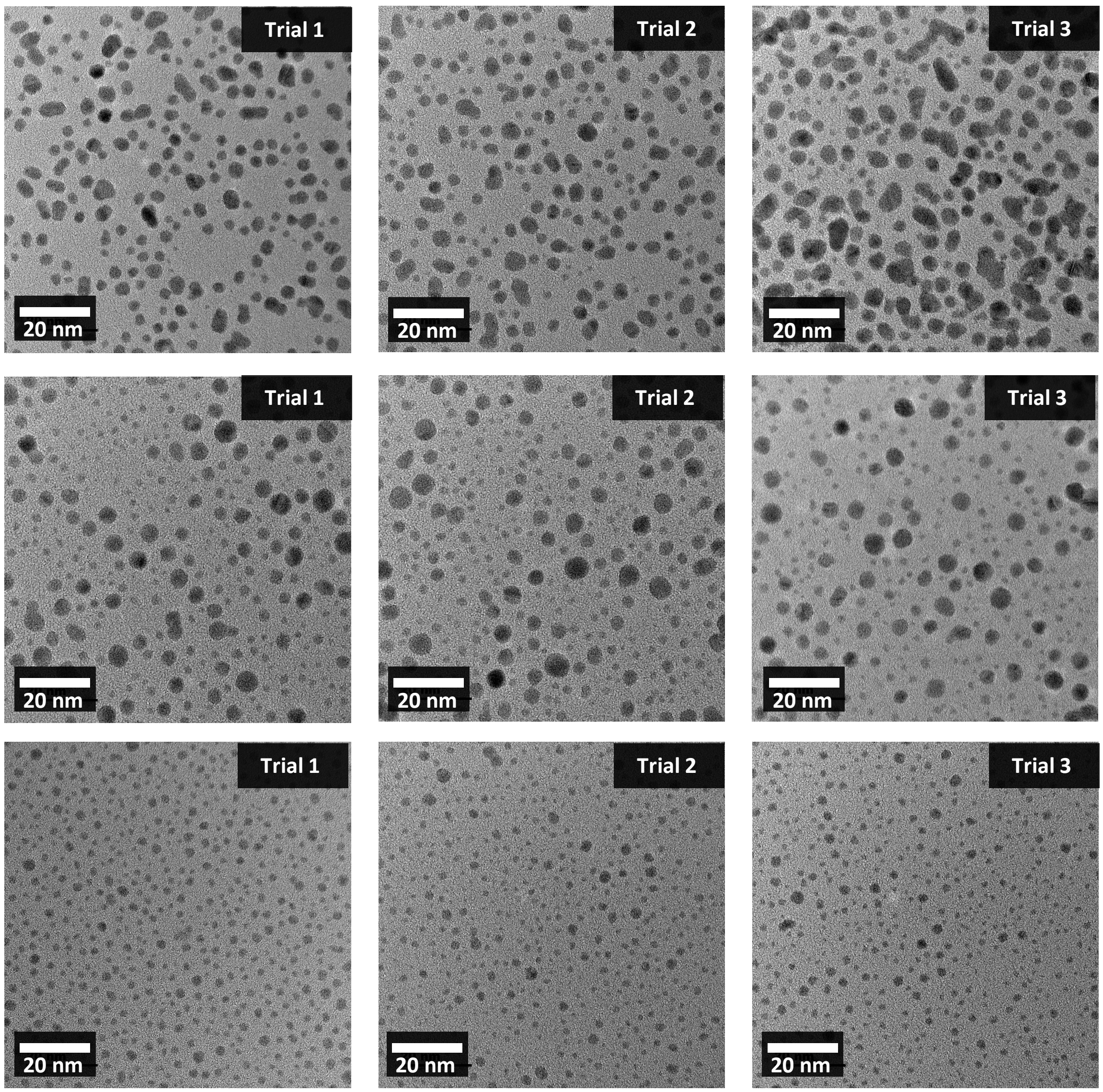

Figure S8. TEM images from three independent synthesis trials for each gold phosphine precursor. Images in the top row correspond to AuTMP, the middle row to AuTEtP, and the bottom row to AuTtBuP, with Trials 1–3 shown from left to right. The images demonstrate the reproducibility of the observed nanoparticle morphologies and relative particle sizes across independent synthesis batches.

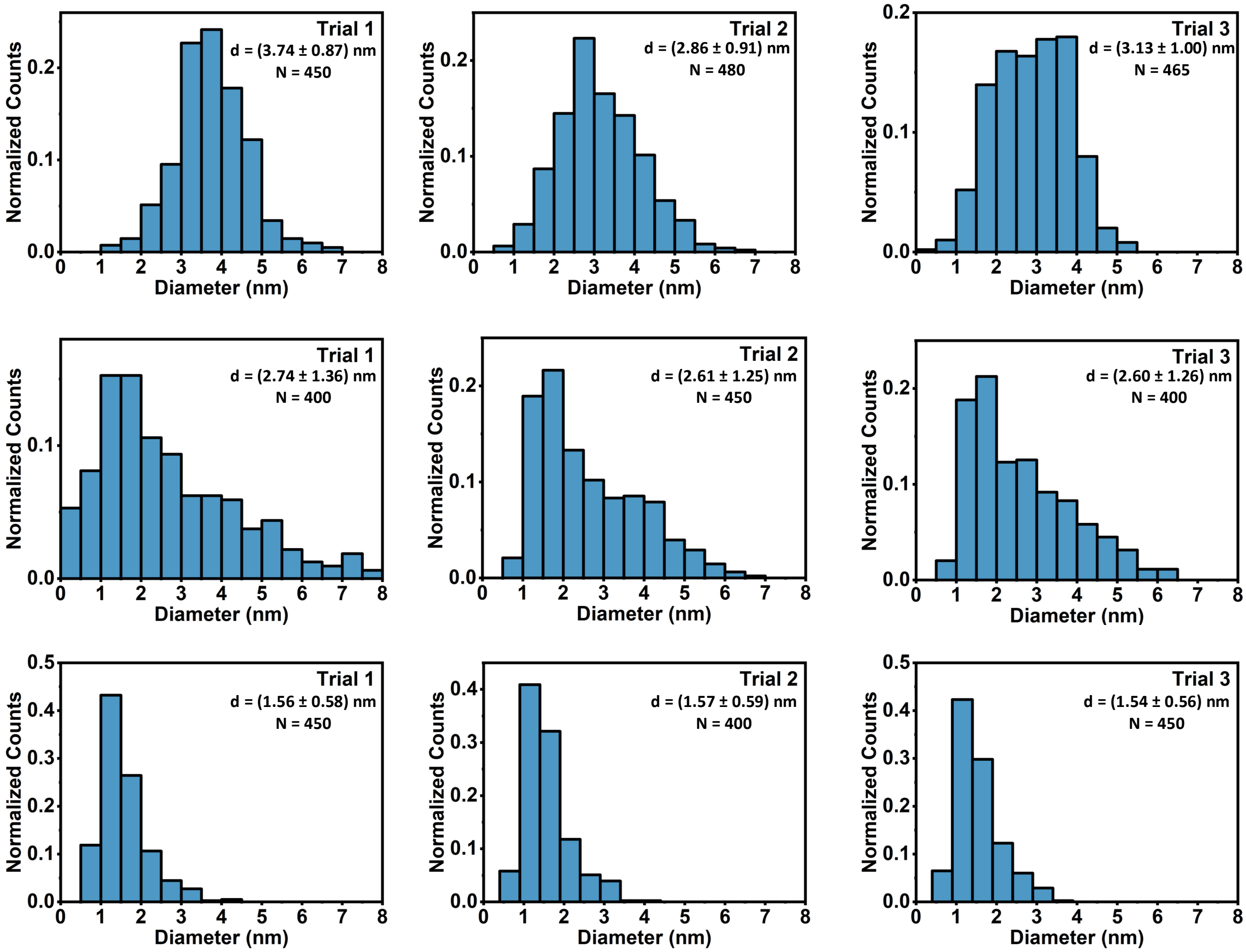

Figure S9. Normalized particle-size distributions obtained from three independent synthesis trials for each gold phosphine precursor. Histograms in the top row correspond to AuTMP, the middle row to AuTEtP, and the bottom row to AuTtBuP, with Trials 1–3 shown from left to right. Counts in each histogram are normalized to the total number of nanoparticles measured in the corresponding trial (relative frequency). "d" represents the mean particle diameter ± within-trial standard deviation, and N represents the total number of nanoparticles measured in that individual trial. The corresponding batch-averaged mean diameters were 3.24 ± 0.45 nm (AuTMP), 2.65 ± 0.08 nm (AuTEtP), and 1.56 ± 0.02 nm (AuTtBuP), where the uncertainty represents the standard deviation of the three independent trial means (n = 3 trials). The particle-size trend AuTMP > AuTEtP > AuTtBuP is maintained across all three independent trials, demonstrating reproducibility of the precursor-dependent size trend from batch to batch.

S6.2 Volume Fraction Calculations

$$\mathrm{VF(small\ particles)} = \frac{\text{Total volume of particles with diameter less than 2 nm}}{\text{Total volume of particles}} \times 100\%$$

$$\mathrm{VF(small\ Particles)} = \frac{\sum_{r=n}^{r<2\ nm}(r_1^3 + r_2^3 + r_3^3 + \cdots)}{\sum_{r=n}^{r<2\ nm}(r_1^3 + r_2^3 + r_3^3 + \cdots) + \sum_{R=n}^{R>2\ nm}(R_1^3 + R_2^3 + R_3^3 + \cdots)} \times 100\%$$

Where r is the radius of a particle smaller than 2 nm, and R is the radius of a particle larger than 2 nm.

REFERENCES

(1) Frisch, M.J., Trucks, G.W., Schlegel, H.B., Scuseria, G.E., Robb, M.A., Cheeseman, J.R., Scalmani, G., Barone, V., Petersson, G.A., Nakatsuji, H., Li, X., Caricato, M., Marenich, A.V., Bloino, J., Janesko, B.G., Gomperts, R., Mennucci, B., Hratchian, H. P., Ortiz, J.V., Izmaylov, A.F., Sonnenberg, J.L., Williams-Young, D., Ding, F., Lipparini, F., Egidi, F., Goings, J., Peng, B., Petrone, A., Henderson, T., Ranasinghe, D., Zakrzewski, V.G., Gao, J., Rega, N., Zheng, G.,Liang, W., Hada, M., Ehara, M., Toyota, K., Fukuda, R., Hasegawa, J., Ishida, M., Nakajima, T., Honda, Y., Kitao, O., Nakai, H., Vreven, T., Throssell, K., Montgomery Jr., J.A., Peralta, J.E., Ogliaro, F., Bearpark, M.J., Heyd, J.J., Brothers, E.N., Kudin, K.N., Staroverov, V.N., Keith, T.A., Kobayashi, R., Normand, J.; Raghavachari, K., Rendell, A.P., Burant, J.C., Iyengar, S.S., Tomasi, J., Cossi, M., Millam, J.M., Klene, M.,

Adamo, C., Cammi, R., Ochterski, J.W., Martin, R. L., Morokuma, K., Farkas, O., Foresman, J.B. and Fox, D.J. Gaussian 16 Rev. C.01 (Wallingford, CT, 2016).

(2) Lee, C.; Yang, W.; Parr, R. G. Development of the Colle-Salvetti Correlation-Energy Formula into a Functional of the Electron Density. Phys. Rev. B **1988**, 37 (2), 785–789. https://doi.org/10.1103/PhysRevB.37.785

(3) Becke, A. D. Density-functional Thermochemistry. III. The Role of Exact Exchange. J. Chem. Phys. **1993**, 98 (7), 5648–5652. https://doi.org/10.1063/1.464913

(4) Grimme, S.; Antony, J.; Ehrlich, S.; Krieg, H. A Consistent and Accurate Ab Initio Parametrization of Density Functional Dispersion Correction (DFT-D) for the 94 Elements H-Pu. J. Chem. Phys. **2010**, 132 (15), 154104. https://doi.org/10.1063/1.3382344

(5) Andrae, D.; Häußermann, U.; Dolg, M.; Stoll, H.; Preuß, H. Energy-Adjusted Ab Initiopseudopotentials for the Second and Third Row Transition Elements. Theor. Chim. Acta **1990**, 77 (2), 123–141. https://doi.org/10.1007/BF01114537

(6) Hehre, W. J.; Ditchfield, R.; Pople, J. A. Self—Consistent Molecular Orbital Methods. XII. Further Extensions of Gaussian—Type Basis Sets for Use in Molecular Orbital Studies of Organic Molecules. J. Chem. Phys. **1972**, 56 (5), 2257–2261. https://doi.org/10.1063/1.1677527

(7) Zhao, Y.; Truhlar, D. G. The M06 Suite of Density Functionals for Main Group Thermochemistry, Thermochemical Kinetics, Noncovalent Interactions, Excited States, and Transition Elements: Two New Functionals and Systematic Testing of Four M06-Class Functionals and 12 Other Functionals. Theor. Chem. Acc. **2008**, 120 (1), 215–241. https://doi.org/10.1007/s00214-007-0310-x

(8) Krishnan, R.; Binkley, J. S.; Seeger, R.; Pople, J. A. Self-consistent Molecular Orbital Methods. XX. A Basis Set for Correlated Wave Functions. J. Chem. Phys. **1980**, 72 (1), 650–654. https://doi.org/10.1063/1.438955

(9) Marenich, A. V.; Cramer, C. J.; Truhlar, D. G. Universal Solvation Model Based on Solute Electron Density and on a Continuum Model of the Solvent Defined by the Bulk Dielectric Constant and Atomic Surface Tensions. J. Phys. Chem. B **2009**, 113 (18), 6378–6396. https://doi.org/10.1021/jp810292n

(10) Pan, P.; Malola, S.; Zhao, R.; Huang, W.; Pohjolainen, E.; Matus, M. F.; Zhou, M.; Kang, X.; Häkkinen, H.; Zhu, M. A Golden Fullerene Encapsulating Schmid Gold. J. Am. Chem. Soc. **2026**, 148 (4), 4579–4587. https://doi.org/10.1021/jacs.5c20164

(11) Balasubramanian, R.; Guo, R.; Mills, A. J.; Murray, R. W. Reaction of Au55(PPh3)12Cl6 with Thiols Yields Thiolate Monolayer Protected Au75 Clusters. J. Am. Chem. Soc. **2005**, 127 (22), 8126–8132. https://doi.org/10.1021/ja050793v

(12) Kenzler, S.; Schrenk, C.; Schnepf, A. Au54(Et3P)18Cl12: A Structurally Related Cluster to Au32(Et3P)12Cl8 Gives Insight into the Formation Process. Dalton Trans. **2020**, 49 (31), 10765–10771. https://doi.org/10.1039/D0DT02262D

(13) Kerdkaew, T.; Limpanuparb, T. The Blue Bottle Experiment Revisited: How Much Oxygen? J. Chem. Educ. **2020**, 97 (4), 1198–1202. https://doi.org/10.1021/acs.jchemed.9b01103

(14) Pons, A. J.; Sagués, F.; Bees, M. A.; Sørensen, P. G. Pattern Formation in the Methylene-Blue−Glucose System. J. Phys. Chem. B **2000**, 104 (10), 2251–2259. https://doi.org/10.1021/jp9935788

(15) Limpanuparb, T.; Areekul, C.; Montriwat, P.; Rajchakit, U. Blue Bottle Experiment: Learning Chemistry without Knowing the Chemicals. J. Chem. Educ. **2017**, 94 (6), 730–737. https://doi.org/10.1021/acs.jchemed.6b00844

(16) Knutson, T. R.; Knutson, C. M.; Mozzetti, A. R.; Campos, A. R.; Haynes, C. L.; Penn, R. L. A Fresh Look at the Crystal Violet Lab with Handheld Camera Colorimetry. J. Chem. Educ. **2015**, 92 (10), 1692–1695. https://doi.org/10.1021/ed500876y

(17) Yusufu, D.; Mills, A. Spectrophotometric and Digital Colour Colourimetric (DCC) Analysis of Colour-Based Indicators. Sens. Actuators B Chem. **2018**, 273, 1187–1194. https://doi.org/10.1016/j.snb.2018.06.131

(18) Keller, M. H.; Moreira, R.; Souza, B. S. Automated Monitoring the Kinetics of Homogeneous and Heterogeneous Chemical Processes Using a Smartphone. Sci. Rep. **2022**, 12 (1), 15774. https://doi.org/10.1038/s41598-022-20123-9
